# Molecular Mechanisms of HMW Glutenin Subunits from 1S^l^ Genome of *Aegilops longissima* Positively Affecting Wheat Breadmaking Quality

**DOI:** 10.1371/journal.pone.0058947

**Published:** 2013-04-04

**Authors:** Shunli Wang, Zitong Yu, Min Cao, Xixi Shen, Ning Li, Xiaohui Li, Wujun Ma, H. Weißgerber, Friedrich Zeller, Sai Hsam, Yueming Yan

**Affiliations:** 1 Key Laboratory of Genetics and Biotechnology, College of Life Science, Capital Normal University, Beijing, China; 2 Institute of Vegetables and Flowers, Chinese Academy of Agricultural Sciences, Beijing, China; 3 State Agriculture Biotechnology Centre, Murdoch University, Western Australian Department of Agriculture and Food, Perth, Western Australia, Australia; 4 Division of Plant Breeding and Applied Genetics, Technical University of Munich, Freising-Weihenstephan, Germany; TGen, United States of America

## Abstract

A wheat cultivar “Chinese Spring” chromosome substitution line CS-1S^l^(1B), in which the 1B chromosome was substituted by 1S^l^ from *Aegilops longissima,* was developed and found to possess superior dough and breadmaking quality. The molecular mechanism of its super quality conformation is studied in the aspects of high molecular glutenin genes, protein accumulation patterns, glutenin polymeric proteins, protein bodies, starch granules, and protein disulfide isomerase (PDI) and PDI-like protein expressions. Results showed that the introduced HMW-GS 1S^l^×2.3* and 1S^l^y16* in the substitution line possesses long repetitive domain, making both be larger than any known x- and y-type subunits from B genome. The introduced subunit genes were also found to have a higher level of mRNA expressions during grain development, resulting in more HMW-GS accumulation in the mature grains. A higher abundance of PDI and PDI-like proteins was observed which possess a known function of assisting disulfide bond formation. Larger HMW-GS deposited in protein bodies were also found in the substitution line. The CS substitution line is expected to be highly valuable in wheat quality improvement since the novel HMW-GS are located on chromosome 1S^l^, making it possible to combine with the known superior D×5+Dy10 subunits encoded by *Glu-D1* for developing high quality bread wheat.

## Introduction

Wheat (*Triticum aestivum* L.) is one of the three most important grain crops in the world. Its flour and dough have unique physical properties for producing bread, cakes, biscuits, pasta and noodles. These unique properties are conferred by the mechanical properties of the gluten proteins mainly consisting of gliadins and glutenins. The glutens enable the retention of CO_2_ in the dough during fermentation, giving its volume to the bread [1]. Gliadins are single chain molecules and impart dough extensibility while glutenins, including high and low molecular weight glutenin subunits (HMW-GS and LMW-GS), consist of protein subunits present in polymers stabilized by interchain disulphide bonds and give dough viscoelasticity [2], [3]. According to previous studies [2], [3], [4], the HMW-GS confers dough strength while the LMW-GS is responsible for dough extensibility. Although HMW-GS only account for about 5–10% of the total proteins in mature seeds, approximately 67% of the variation of baking parameters among wheat cultivars can be attributed to differences in HMW-GS compositions [5]–[8].

It is known that HMW-GS are encoded by *Glu-1* loci on the long arms of the homeologous group 1 chromosomes 1A, 1B and 1D, while LMW-GS were encoded by *Glu-3* loci on the short arms of the same chromosomes [9]. For *Glu-1*, there are two closely linked genes (*Glu-1–1* and *Glu-1*–*2*) at each of the three loci, *Glu-A1*, *Glu-B1* and *Glu-D1*, in common wheat, which encode one larger x-type (80–88 KDa) and one smaller y-type (67–73 KDa) subunit, respectively [10]. The allelic variation of HMW-GS at each locus is associated with the end-use product quality in bread wheat [11]. So far, numerous HMW-GS have been identified and characterized, and the significant relationships between some subunits and quality attributes have been found [12]. For example, 1Dx5 +1Dy10 and 1B×17+1By18 are associated with high gluten elasticity and strong dough characteristics suitable for good quality breadmaking whereas 1Dx2+1Dy12 and 1B×20 have negative effects on gluten quality [2], [11], [13]–[15].

The molecular structures of HMW glutenin subunits have four primary regions, the signal peptide (removed from mature protein), N- and C-terminal domains, and a central repetitive region [11]. The protein variations and size differences among different HMW-subunits are mainly caused by variations in the length of the repetitive domains, which contain different numbers of repeated peptide motifs [16]. Although a great number of studies have been conducted in the past several decades, the molecular mechanisms of HMW-GS allelic variations on wheat breadmaking quality are still an active research topic. Until now, a number of factors are believed to be important, including the number and position of cysteine residues [17], [18], the molecular structural features of repetitive domains [19], [20], the presence of chain terminators [21], the gluten protein compositions and polymer distributions in the mature grains [22], and the over-expression of certain subunits such as B×7^OE^ as well as the accumulation rates of HMW-GS during grain development [22], [23]–[25]. It is generally accepted that the HMW-GS genes with longer repetitive domains, higher expression amount and having extra cysteines for better chain extension may result in good quality of wheat [21]. Recent studies by an in *vitro* assay showed that x- and y- type subunits incorporated together gives synergistic effects on dough parameters than those with either subunit type alone [26], [XPATH ERROR: unknown variable "next".].

To date, extensive investigations have demonstrated that high levels of allelic variations at *Glu-1* loci are highly sought after in the quality wheat breeding practice [12]. *Aegilops* and other wheat related species, has rich HMW-GS variations and may provide potential quality gene sources to meet the demand of improving wheat quality [28]–[33]. The genus *Aegilops* contains 22 species with S, M, C, U, N and T genomes. The S genome, which is considered as the progenitor of bread wheat B genome, constitutes a group of related species in the section *Sitopsis* that includes *Ae. speltoides* (S), *Aegilops bicornis* (S^b^), *Aegilops longissima* (S^l^), *Aegilops searsii* (S^s^), and *Aegilops sharonensis* (S^sh^). Although a number of HMW glutenin subunits in *Sitopsis* have been identified and characterized [30], [34], their effects on breadmaking quality have not been investigated. Particularly, little is known about the functional properties of HMW-GS from *Aegilops longissima*.

We produced a Chinese Spring (CS) substitution line in which a chromosome (1S^l^) from *Aegilops longissima* substituted one chromosome (1B) of CS with a significantly increase of dough rheological property. In the current study, we thoroughly characterized the mechanisms of this substitution line in aspects of high molecular glutenin coding genes, protein accumulations, protein bodies, starch granules and protein disulfide isomerase (PDI) and PDI-like protein expressions (PDI and PDI-like protein can control diversified metabolic functions) in order to uncover the mechanism underlying the superior dough and breadmaking quality of this substitution line.

## Results

### Characterization of Chinese Spring substitution line and its quality performance

The development of Chinese Spring substitution line CS-1S^l^(1B) was showed in Figure S1A, and its chromosome compositions were identified by GISH using *Ae. longissima* genomic DNA as probe. The result showed that the 1S^l^ chromosome successfully substituted the 1B chromosome of CS (Figure S1B). The morphological characterizations of plants, spikes and seeds as well as growth and development traits between CS and CS-1S^l^(1B) displayed higher similarity (Figure S1C and Table S1).

**Figure 1:**
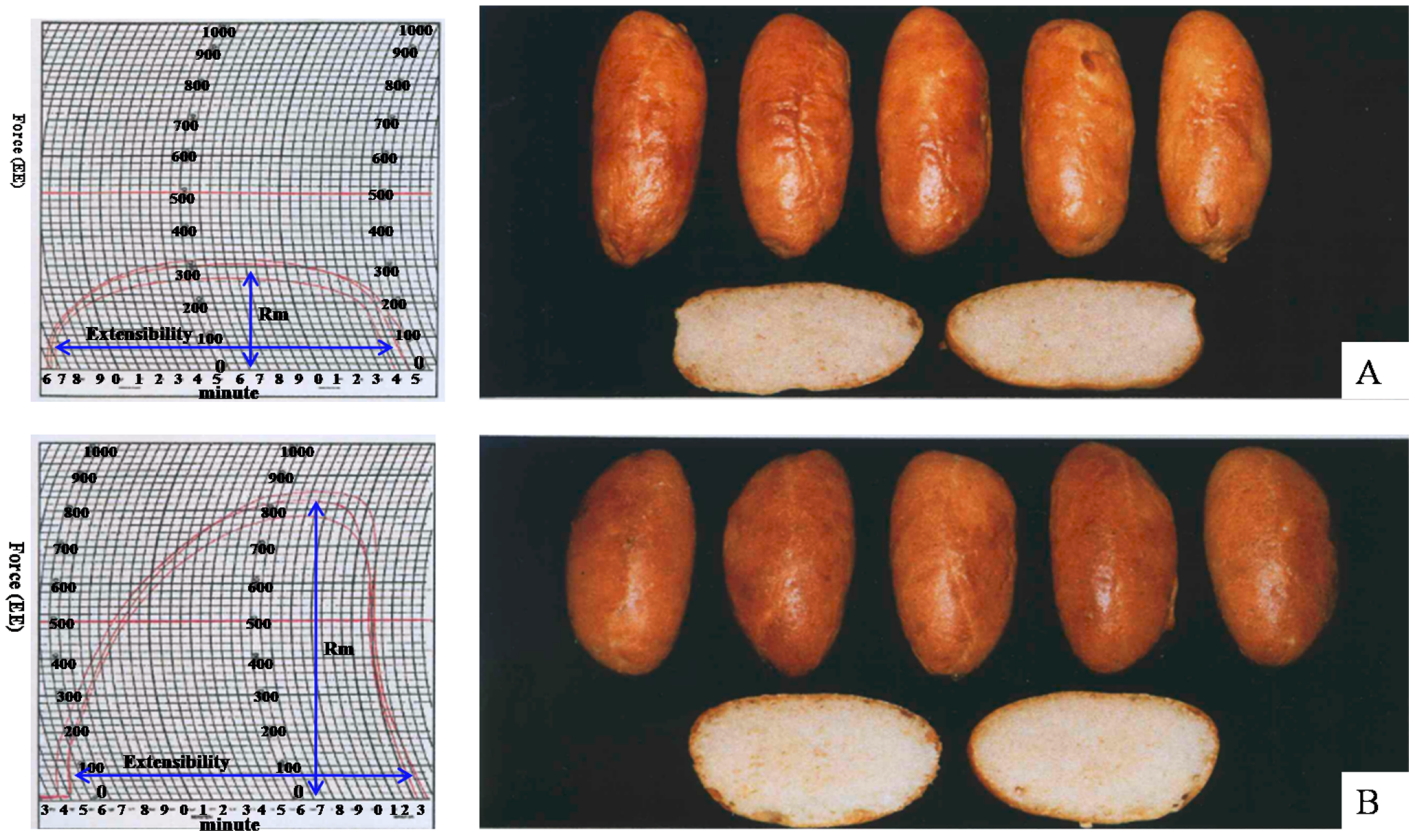
Extensogram (left) and loaves baking (right) in CS (A) and and substitution line CS-1S^l^(1B) (B). EE represents Extensogram Einheit (it is the fore unit used in German), Rm represents resistance maximum.

Flour quality analysis demonstrated that both dry and wet glutens as well as the SDS-sedimentation value in CS-1S^l^(1B) were higher than those in CS as shown in Table 1. The 1S^l^(1B) substitution line significantly altered the Mixograph properties of base flour and significantly increased the peak time and peak width at 8 min, compared with CS. Extensograph results revealed that the dough maximum resistance (Rmax) of CS-1S^l^(1B) increased more than 2 times. However, no significant difference of dough extensibility was observed between two lines. Baking parameters including tearing- and extension surface, tearing- and extension length and maximum height were measured to evaluate two lines's breadmaking quality (Table 1). Results demonstrated that CS-1S^l^(1B) had large extension surface with an average value of 128.4 cm^2^ while CS only had 42 cm^2^. Particularly, the loaf volume as measured by the rapid-mix-test significantly increased 27.1%, from 550 cm^3^ in CS to 699 cm^3^ in CS-1S^l^(1B) as shown in Table 1 and Figure 1.

**Table 1:** Quality parameters of CS and CS-1S^l^(1B).

Materials	Ash (%)	Wet glutenin (%)	Dry glutenin (%)	Peak time (min)	Peak Integral (%tq×min)	Width at 8 min (mm)	SDS-sedimentation value (ml)	Loaf volume (cm^3^)
CS	0.67±0.02	17±0.26	19.4±0.21	1.8±0.03	67.65±0.32	3.25±0.20	45±0.62	550±1.18
CS-1S^l^(1B)	0.70±0.03	17.5±0.30	19.9±1.11	2.1±0.06	76.95±0.39	3.85±0.20	65±0.44	699±1.73
Materials	Water absorption (%)	Extension surface (cm^2^)	Extension length (mm)	Tearing surface (cm^2^)	Tearing length (mm)	Maximum height (EE)	Extension length ratio	GMP (%)
CS	62.3±0.53	42.0±0.44	107.6±0.70	32±0.56	71.0±0.52	298±4.13	1.51±0.03	3.18±0.02
CS-1S^l^(1B)	58.7±1.15	128.4±0.85	132.6±0.40	55±1.57	42.3±0.26	821±10.67	3.13±0.03	3.39±0.60

(Mean value of 3 replicates and the standard deviation).

### Identification of seed proteins in Chinese Spring and 1S^l^(1B) substitution line

**Figure 2:**
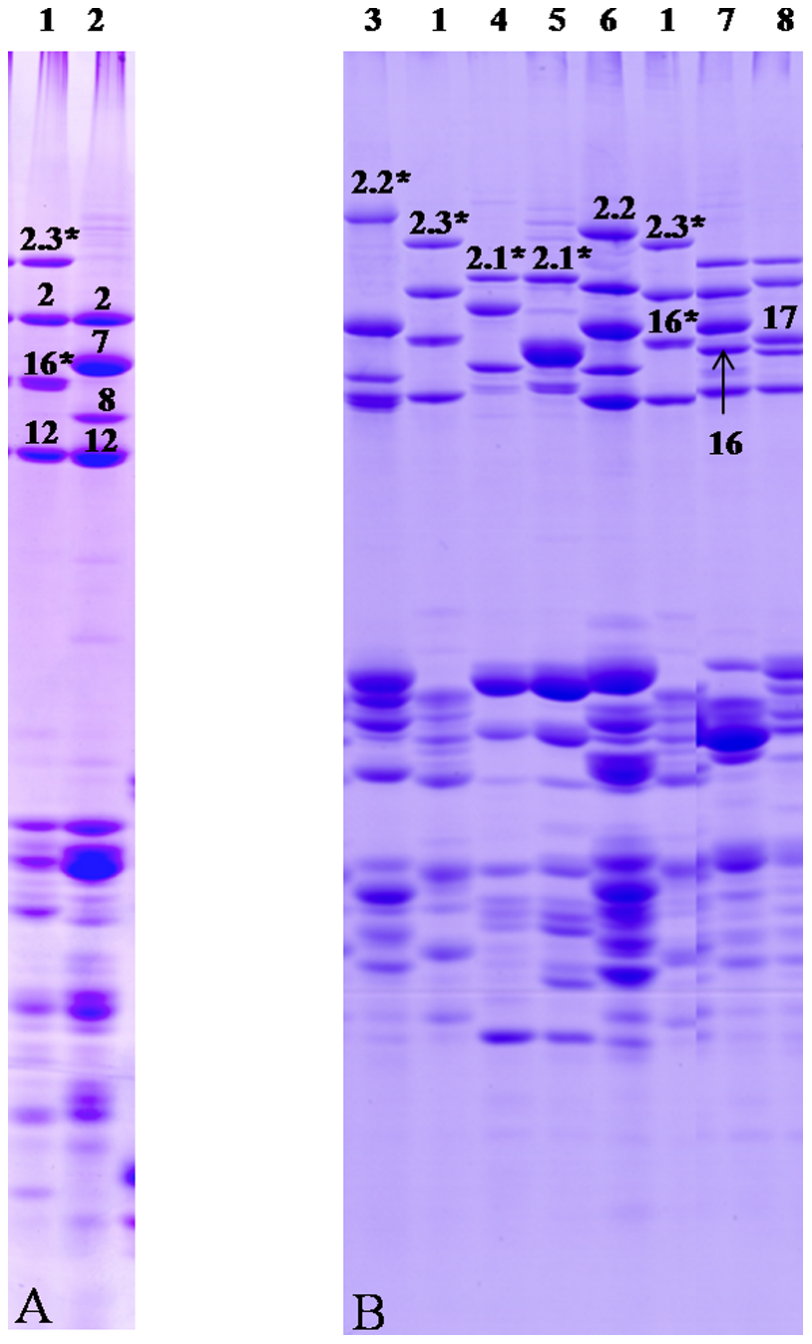
HMW and LMW glutenin subunits of CS and CS-1S^l^(1B) identified by SDS-PAGE (A), and comparison with standard cultivars (B). 1: CS-1S^l^(1B) (N, 1S^l^x2.3*+1S^l^y16*, 1Dx2+1Dy12), 2: CS (N, 1Bx7+1By8, 1Dx2+1Dy12), 3: MG315 (N, 1Bx7+1By8, 1Dx2.2*+1Dy12), 4: Dico140 (1Ax2.1*, 1Bx6+1By8), 5: Dico17 (1A×2.1*, 1Bx17*+1By18*), 6: MG7249 (1A×2*, 1Bx7+1By8, 1Dx2.2+1Dy12), 7: Jimai 20 (1Ax1, 1Bx13+1By16, 1Dx4+1Dy10), 8: CB037 (1A×1, 1B×17+1By18, 1D×2+1Dy12).

**Figure 3:**
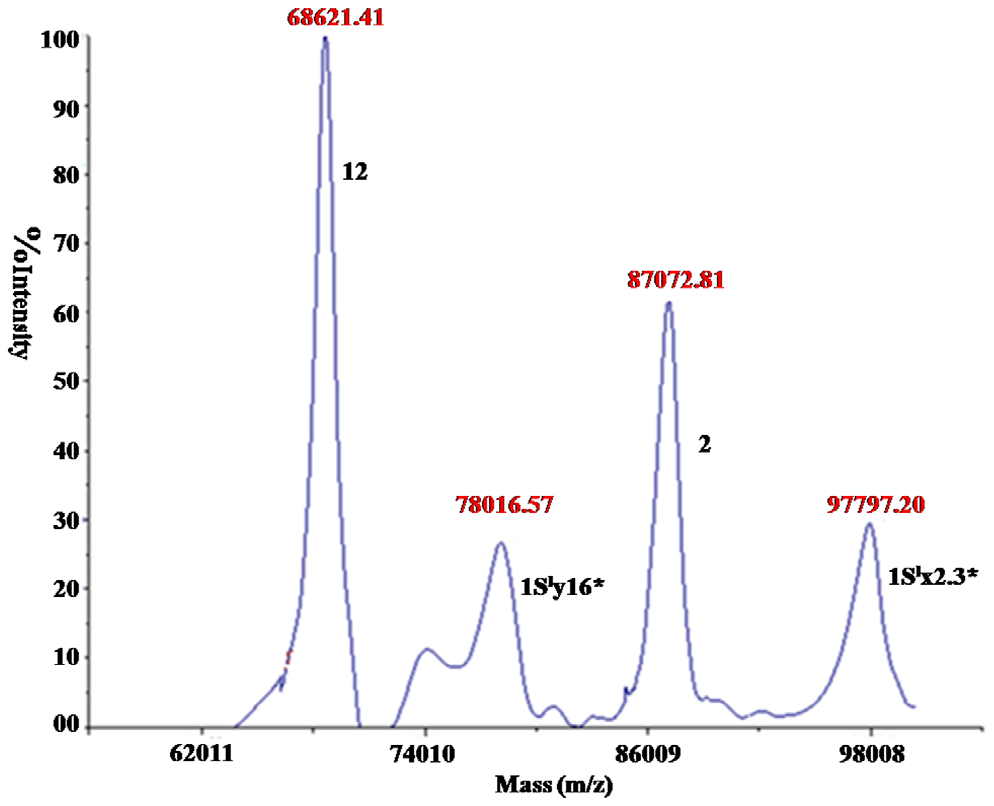
HMW-GS in CS-1S^l^(1B) identified by MALDI-TOF-MS. 1S^l^×2.3* and 1S^l^y16* subunits and their accurate molecular mass were indicated.

SDS-PAGE indicated that two novel HMW-GS subunits were present in CS-1S^l^(1B) but without the 1B-encoded 1Bx7+1By8 subunits (Figure 2A), demonstrating that two 1S^l^-encoded novel subunits replaced two 1B-encoded subunits. The electrophoretic mobilities of 1S^l^-encoded x- and y-type subunits were close to those of 1D×2.2 and 1By16, respectively (Figure 2B), and therefore designated as 1S^l^×2.3* and 1S^l^y16*. RP-HPLC analysis failed to separated 1S^l^×2.3* and 1S^l^y16* from 1D×2 and 1Dy12 subunits, respectively (Figure S2A), suggesting that both x- and y-type subunit pairs had similar hydrophobicity although their molecular weights were significantly different. RP-UPLC well separated 1S^l^y16* from 1Dy12 subunits, but 1S^l^×2.3* and 1Dx2 subunits were still inseparable (Figure S2B). Accurate molecular mass of 1S^l^×2.3* and 1S^l^y16* was determined by MALDI-TOF-MS. As shown in Figure 3, two separated mass spectra with *M*_r_ 97797 Da and 780165 Da were present in the high molecular mass region, well corresponding to the positions of 1S^l^×2.3* and 1S^l^y16* subunits in the SDS-PAGE gel, respectively. These results indicates that the introduced x-type subunit 1S^l^×2.3* is larger than any known 1B-encoede x-type subunits while 1S^l^y16* is the largest among all y-type subunits identified in *Triticum* species so far.

The compositions of LMW-GS, gliadins, albumins and globulins in CS and CS-1S^l^(1B) were shown in Figure S2–3. RP-HPLC and SE-HPLC revealed some differences in LMW-GS and gliadin compositions between CS and CS-1S^l^(1B), resulting from 1S^l^(1B) substitution (Table 2 & Figure S2A–B). SE-HPLC quantification results of total protein, gliadin, glutenin protein in CS and CS-1S^l^(1B) were 30714.7 (AU)^b^ and 38200.3 (AU)^b^, 9993 (AU)^b^ and 10273 (AU)^b^, 16382.4 (AU)^b^ and 21736.3 (AU)^b^, respectively (Figure S9). The gliadin/glutenin ratios in CS and CS-1S^l^(1B) were 0.610 and 0.473, respectively. The total peak areas of HMW-GS, LMW-GS and alpha/beta-gliadin, gamma-gliadin, omega-gliadin in CS were 20459.20, 30786.20, 215463, 45939 and 90920 mAU*min (mini-Absorb unit * minute), respectively. While in the substitution line, the total peak areas of the five proteins were 34733.4, 38317.4, 184347, 31410 and 79710 mAU*min. The HMW-GS/LMW-GS ratios in CS and CS-1S^l^(1B) were 0.660 and 0.897, respectively. However, their albulmin and globulin compositions displayed higher similarity (Figure S3). Since HMW-GS are major determinant for dough elasticity, the introduced 1S^l^×2.3* and 1S^l^y16* subunits could be responsible for the superior breadmaking quality of substitution line CS-1S^l^(1B).

**Table 2:** HPLC results of Chinese Spring (CS) and CS-1S^l^(1B).

SE-HPLC						RP-HPLC					
	Total protein (AU)^b^	Gliadin (AU)^b^	Glutenin (AU)^b^	Gliadins/Glutenin	HMW-GS (mAU*min)		LMW-GS (mAU*min)	alpha/beta-gliadin (mAU*min)	gamma-gliadin (mAU*min)	omega-gliadin (mAU*min)	HMW-GS/LMW-GS
CS	30714.7	9993	16382.4	0.610	20459.20		30786.20	215463	45939	90920	0.660
CS-1S^l^(1B)	38200.3	10273	21736.3	0.473	34733.4		38317.4	184347	31410	79710	0.897

### HMW-GS determination by MALDI-TOF/TOF-MS

In order to further verify the presence of the two novel 1S^l^-encoded HMW-GS in CS-1S^l^(1B) and obtain their structural information, both 1S^l^×2.3*+1S^l^y16* subunits from SDS-PAGE gel were excised manually. After in-gel trypsin digestion, MALDI-TOF/TOF-MS analysis was performance. The results showed that the 1S^l^×2.3* and 1S^l^y16* subunits were identified as HMW-GS gi|47834185 (x-type HMW-GS from *Aegilops bicornis*) and gi|24474920 (y-type HMW-GS in *T. aestivum*), respectively (Table S2). Three peptides between 1S^l^×2.3* and gi|47834185 and six peptides between 1S^l^y16* and gi|24474920 were matched with protein score C.I. % of 100%.

### Molecular characterization of 1S^l^-encoded HMW-GS genes

The complete open reading fragms (ORFs) encoded 1S^l^×2.3* and 1S^l^y16* subunits were isolated and cloned from CS-1S^l^(1B) by AS-PCR. The full length ORFs of 1S^l^x2.3* and 1S^l^y16* were 2829 bp and 2250 bp, encoding 941 and 749 amino acid residues, respectively. Their deduced molecular weights (97851 Da and 78118 Da) were well consistent with those determined by MALDI-TOF-MS (97797 Da and 780165 Da), suggesting no post-translational modifications present in both subunits.

Comparison analysis of the deduced protein sequences showed that 1S^l^×2.3* had typical characteristics of x-type HMW-GS, including a signal peptide of 21 amino acid residues, an N-terminal domain of 86 amino acid residues, followed by a repetitive domain of 792 amino acid residues and a C-terminal domain of 42 amino acid residues (Figure S4). The repetitive domain of 1S^l^×2.3* contained 31 hexapeptides (consensus PGQGQQ and SGQGQQ), 11 nonapeptides (consensus GYYPTSPQQ and GYYPTSLQQ) and 24 tripeptide (consensus GQQ) motifs. Four cysteine residues were distributed at conserved positions as other x-type HMW-GS: three in the N-terminal domain (at positions 31, 43 and 58) and one in the C-terminal domain (at position 929). Our results demonstrate that 1S^l^x2.3* is the largest HMW-GS among the HMW-GS x-type subunits encoded by 1B. Among all reported HMW-GSs, it is only a little smaller than two subunits on the D genome, 1Dx2.2 (2919 bp) and 1Dx2.2* (3078 bp) [35].

1S^l^y16* appeared to be the largest y-type HMW-GS in *T. aestivum* (Figure S5) since it is larger than the previously reported largest y-type subunit, 1By16 (2220 bp) [14]. It contained a signal peptide of 21 amino acid residues, an N-terminal domain of 104 amino acid residues, followed by a repetitive domain of 582 amino acid residues and a C-terminal domain of 42 amino acid residues. The repetitive domain contains 25 hexapeptide (consensus PGQGQQ and SGQGQQ) and 8 nonapeptide (consensus GHYPASQQQ or GYYPTSLQQ) motifs. Seven cysteine residues were present at conserved positions as other y-type subunits: three in the N-terminal domain (at positions 31, 43, and 65, 66 and 76), one in the repetitive domain (at position 635) and one in the C-terminal domain (at position 737).

The coding sequences of 1S^l^×2.3* and 1S^l^y16* subunit genes were aligned with other 17 x-type and 17 y-type HMW-GS genes, respectively. The SNPs and InDels present in the two 1S^l^-encoded HMW-GS genes were identified (Table 3–4). A total of 11 SNPs were detected at different positions in each gene while 4 InDels were present in 1S^l^x2.3* and only one deletion was detected in 1S^l^y16*. Apparently, a 97 bp insertion resulted in the increase of 1S^l^x2.3* gene size. In addition, more than half of the detected SNPs were nonsynonymous in both genes.

**Table 3:** the positions of SNPs and InDels identified in *1S* ^l^ x2.3*.

HMW-GS	285	350	543	679	862–870	1741	1769	2194–2211	2374–2471	2530	2608	2733	3553	3622	3628
1Slx2.3*	A	G	T	G	---------------	T	A	TACTACCCAACTTCTCTG	CAACAACCAGGACAAGGGCAACAAGGGCAGCAA CCAGCACAAGGGCAACGAGGTCAACAGCCAACA CAAGGGCAACGAGGTCAGCAGCCAGCACAAGG	G	C	C	T	T	G
Other 17 HMW-GS Genes1	G	C	C	A	GGGTACTAC	G	G	-----------------------------------	----------------------------------------------------------------------	C	A	-	C	C	A

1 Other 17 HMW-GS genes were from GenBank, including HQ380225 (*Ae. speltoides*), HQ380224 (*Ae. speltoides*), GQ403043 (*Ae. longissima*), AY611723 (*Ae. searsii*), AY611727 (*Ae. bicornis*), X13927 (*T. aestivum*), EF540764 (*T. aestivum*), AY367771 (*T. aestivum*), AB263219 (*T. aestivum*), AJ437000 (*T. turgidum* subsp. *durum*), AY553933 (*T. aestivum*), X61009 (*T. aestivum*), X12928 (*T. aestivum*), AY455789 (*Ae. comosa*), AF476959 (*Ae. markgrafii*), AY455786 (*Ae. uniaristata*) and AF476961 (*Ae. umbellulata*).

**Table 4:** the positions of SNPs and InDels identified in *1S* ^l^ y16*.

HMW-GS	36	38	56	165	1088	1225	1346	1917	2157	2383–2400	2554–2555
*1S* ^l^ y16*	A	C	G	T	C	A	T	C	G	--------------------------------------	AT
Other 17 HMW-GSGenes^1^	C	T	C	G	A	C	C	A	A	CAAGGGCAGCAATCAGGA	TC

1 Other 17 HMW-GS genes were from GenBank, including HQ380222 (*Ae. speltoides*), HQ380223 (*Ae. speltoides*), HQ380229 (*Ae. kotschyi*), HQ380227 (*Ae. kotschyi*), HQ380228 (*Ae. kotschyi*), AY245797 (*T. turgidum* subsp. *durum*), X61026 (*T. aestivum*), DQ086215 (*T. aestivum*), EF540765 (*T. aestivum*), AF476960 (*Ae. t markgrafii*), AF476962 (*Ae. umbellulata*), AY611728 (*Ae. bicornis*), AY611724 (*Ae. searsii*), GQ403044 (*Ae. longissima*), AY455788 (*Ae. comosa*), AY455787 (*Ae. uniaristata*) and X12929 (*T. aestivum*).

### In vitro expression and identification of 1S^l^ encoded HMW-GS

To further verify the authenticity of the cloned 1S^l^-encoded HMW glutenin genes, the ORF sequences of both genes were expressed in the bacterial cells after removing the signal peptide by PCR mutagenesis. After cloning the modified ORFs into the pET-28a vector, the recombinant 1S^l^x2.3* and 1S^l^y16* genes (pET-2.3* and pET-16*) were expressed in *E. coli*. The expressed proteins were purified by 50% (v/v) propanol containing 1% DTT (w/v) and separated by SDS-PAGE (Figure S6). Results showed that the electrophoretic mobilities of the two expressed proteins were similar to the native 1S^l^-encoded 1S^l^x2.3* and 1S^l^y16* subunits in CS-1S^l^(1B). The authenticities of the expressed proteins were then verified by Western blotting. The bacterially expressed proteins and the endogenous HMW-GS subunits of CS-1S^l^(1B) both displayed a strong reaction to the polyclonal antibody specific for HMW glutenin subunits (Figure S6).

### Phylogenetic evolutionary analysis of 1S^l^-encoded HMW-GS genes

A phylogenetic tree was constructed based on the complete amino acid sequences of 43 HMW-GS genes from *Aegilops* and *Triticum* species by MEGA4.1. As shown in Figure S7, the HMW-GS genes were clustered into 2 separated branches, corresponding to x-type and y-type HMW-GS genes. The estimated divergent time between x-type and y-type HMW-GS genes was 21.089 million year ago (MYA), which is similar to the previous reports [30], [34]. 1S^l^×2.3* and 1S^l^y16* had a higher similarity with HQ380226 and AY611728 subunits from *Aegilops kotschyi* and *Aegilops bicornis*, respectively. Most of the HMW-GS encoded by the genes from 1B and 1S, 1S^s^, 1S^b^ genomes were clustered together, indicating their close relationships. However, both 1S^l^×2.3* and 1S^l^y16* fell into the HMW-GS subgroup encoded by D genome (Figure S6), indicating that 1S^l^-encoded HMW-GS had higher sequence similarity with those encoded by the D genome.

### Transcriptional expression analysis of HMW-GS and PDI genes during grain development

**Figure 4:**
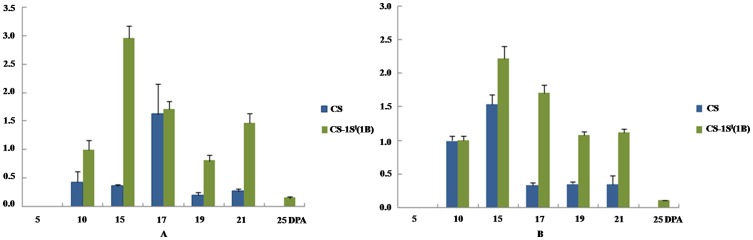
Expression patterns of HMW-GS genes in CS and CS-1S^l^(1B) by qRT-PCR. A. x-type HMW-GS genes; B. y-type HMW-GS genes.

In general, x- and y-type HMW-GS genes showed a similar expression trend in CS and CS-1S^l^(1B) (Figure 4) and both types expressed much lower at 5 DPA, but rapidly up-regulated at 10 DPA. However, their transcriptional expression levels in CS-1S^l^(1B) were higher than those in CS during all grain development stages. The maximum expression level in CS-1S^l^(1B) occurred at 15 DPA. The expression of y-type HMW-GS genes in CS and CS-1S^l^(1B) was gradually up-regulated from 5 to 15 DPA, and then down-regulated from 17 to 21 DPA, which was similar to the x-type HMW-GS genes in CS-1S^l^(1B). However, the up-regulated expression of the x-type HMW-GS genes in CS was from 5 to17 DPA. Both HMW-GS gene types displayed a much lower level at 25 DPA. In the substitution line, both genes expressed in a higher level and longer time than that in CS. Based on these results, it can be concluded that the introduced HMW-GS in the substitution line have higher and longer expression at the mRNA level.

**Figure 5:**
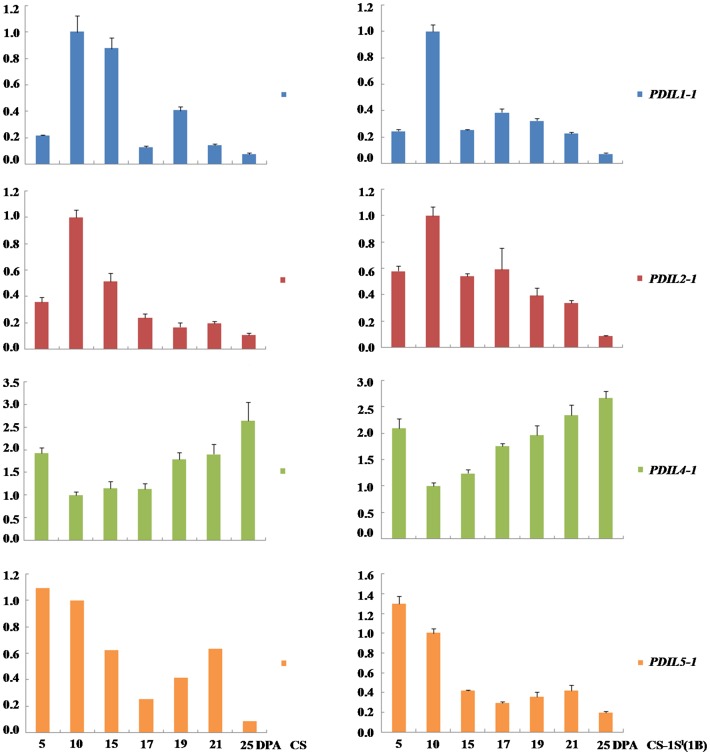
Expression profiles of four groups of PDI and PDI like genes (*PDIL1-1*, *2-1*, *4-1* and *5-1*) in CS and CS-1S^l^(1B) by qRT-PCR.

Protein disulfide isomerase (PDI) gene family encodes several PDI and PDI-like proteins containing thioredoxin domains, and is involved in the correct formation of disulphide bond, protein folding and assembling of protein bodies [36], [37]. d'Aloisio *et* *al*. [37] classified PDI and PDI-like sequences cloned from wheat into eight groups by phylogenetic analysis. In this work, the expression patterns of these eight group genes in seven grain developmental stages were analyzed by qRT-PCR. The analyzed caryopses covered the stages of endosperm development of cellularisation to desiccation. Previous studies showed that the proteins encoded by genes in groups I, II, IV and V were involved in the folding of storages proteins in soybean [38]–[41]. To study the relationship between PDIs and HMW-GS in wheat, the expression patterns of PDIs in all eight groups in seven grain developmental stages were analyzed by qRT-PCR (Figure 5 and S8). The results of the expression patterns of *PDIL1-1*, *PDIL2-1*, *PDIL4-1* and *PDIL5-1* revealed a considerable variation of transcription expression levels among these four gene groups. The expression patterns of typical PDI (*PDIL1-1*) and *PDIL2-1* genes were similar in CS and CS-1S^l^(1B). Both genes were initially detected at 5 DPA and reached the highest level at 10 DPA, then decreased. The expression of *PDIL1-1* had a slight increase at 19 DPA in CS and at 17 DPA in CS-1S^l^(1B), but decreased in both CS and CS-1S^l^(1B) at the late grain development stages (Figure 5). An increased expression of *PDIL2-1* in CS and CS-1S^l^(1B) occurred at 17, 21 and 17 DPA, respectively. *PDIL4-1* and *PDIL5-1* also displayed a higher expression level in the early grain development stage (5 DPA), and then decreased gradually. After 10 DPA, the expression of *PDIL4-1* was increased again, reaching the highest level at the maturity in two lines (Figure 5). There was a higher expression of *PDIL4-1* in the substitution line than that of CS. In contrast, the expression of *PDIL5-1* showed a down-up-down pattern during grain development stages.

The expression of the other four groups of PDI and PDI-like protein genes displayed multiple patterns during grain development. The *PDIL3-1* showed a much high expression level at 5 DPA, and then decreased at 10 DPA. Subsequently, it increased from 15 to 17 DPA, and remained the high level until 25 DPA (physiological maturity). Its expression level in CS-1S^l^(1B) was higher than that in CS. The expression pattern of *PDIL6-1* was very similar to that of the *PDIL3-1* during the late stage of seed development, especially in CS–1S^l^(1B), but they had different expression patterns in the early grain development stage (Figure S8). In addition, the expression pattern *PDL7-1* and its paralogous gene *PDIL7-2* had no significant difference. Both genes had an increase from 5 to 15 DPA in CS, while they had a little decrease at 10 DPA in the substitution line and maintained a high level until maturity. In CS–1S^l^(1B), their expression was relatively low during 5 to 15 DPA but had a sharp increase after 15 DPA and constantly kept the high expression level until maturity. The expression pattern of *PDIL8-1* was similar in CS and CS–1S^l^(1B), displaying a higher level at 5 DPA and decreased rapidly at 10 DPA, then increased at 17 DPA, subsequently decreased until maturity.

### Synthesis and accumulation patterns of HMW-GS during grain filling

**Figure 6:**
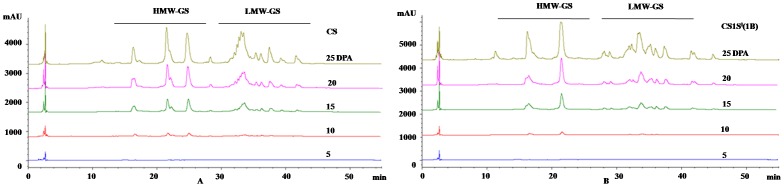
Synthesis and accumulation patterns of glutenins during different grain developmental stages in CS (A) and CS-1S^l^(1B) (B) by RP-HPLC.

RP-HPLC analysis of the synthesis and accumulation characteristics of HMW-glutenins in CS and CS-1S^l^(1B) demonstrated that glutenin proteins synthesis initiated at 10 DPA and then steadily increased until grain maturity (Figure 6). The important stages for HMW-glutenin synthesis were 15–20 DPA during which the HMW-glutenin proteins accumulated to a considerable amount. Compared with CS, CS–1S^l^(1B) expressed a higher HMW-glutenin amount from 10 to 25 DPA and finally reached the highest amount at 25 DPA. The GMP content result showed that it was higher in CS–1S^l^(1B) (3.39%) than that in CS (3.18%). This suggested that high amount of glutenin polymeric protein requires a high HMW-GS accumulation during grain development. Quantification of total protein, gliadin, glutenin protein by SE-HPLC in CS and CS-1S^l^(1B) were 30714.7 (AU)^b^ and 38200.3 (AU)^b^, 9993 (AU)^b^ and 10273 (AU)^b^,16382.4 (AU)^b^ and 21736.3 (AU)^b^, respectively (Figure S9). And the ratios of gliadins/glutenins in CS and CS–1S^l^(1B) were 0.610 and 0.473, respectively. The accumulation pattern of LMW-GS was similar between CS and CS–1S^l^(1B), which had a stable increase based on the results of RP-HPLC (Figure 6). Taking together with the results from qRT-PCR, CS–1S^l^(1B) had higher HMW-GS expression amounts than CS in both transcriptional and translational levels.

### Observation of dense protein bodies (PB) from developing grains

**Figure 7:**
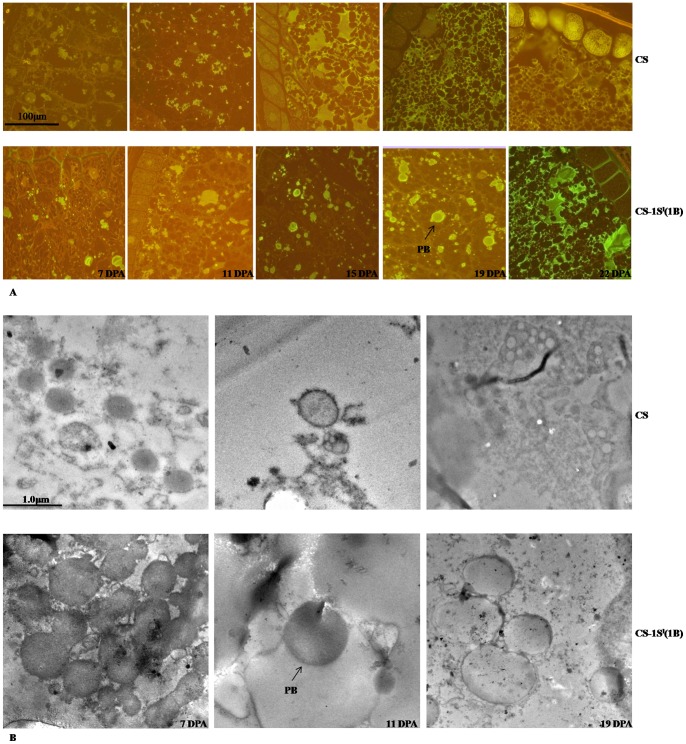
Fluorescence microscopy and electron micrographs of protein bodies (PB) in the endosperm cells during developing grains. A. Light micrographs of wheat grain developing endosperm at 7, 11, 15, 19 and 22 DPA by fluorescent labellings. B. Electron micrographs of ultrathin sections of wheat grain developing endosperm.

Wheat seed storage proteins deposited into organelles of protein bodies (PBs) after synthesis and folding in the lumen of the endoplasmic reticulum (ER) [42], [43]. Fluorescence microscopy analysis showed that both CS and CS–1S^l^(1B) had similar morphologies of the endosperm cells and similar ontogeny of PBs. The PBs immunolabelled with anti-HMW-GS were detected at 7 DPA in both lines; they grew in size by fusion among themselves and finally reached the maximum size at 15 and 19 DPA in CS and CS–1S^l^(1B), respectively (Figure 7A). At 15 DPA, microscopy observations demonstrated that PBs did not remain as separate particles but coalesced to form large aggregates in CS (Figure 7A), whereas in CS–1S^l^(1B) the PBs were kept getting larger from 15 DPA to 19 DPA. At 19 DPA and 22 DPA, no PBs could be observed by fluorescence microscopy in CS, while large and normal PBs could be detected in CS-1S^l^(1B). These suggest that the HMW-GS in CS began to form a matrix entrapping starch granules from 19 DPA and 22 DPA in CS and CS–1S^l^(1B), respectively. The average area of PBs labeled with anti-HMW-GS in CS–1S^l^(1B) was much more than those in CS (Figure 7A and Table S3). This suggests that more HMW-GS was accumulated in CS–1S^l^(1B) than that in CS.

The PB sizes were studied by TEM experiments on endosperm sections at 7, 11 and 19 DPA. At 7 DPA, the diameter of PB was 0.4–0.5 μm in CS and 0.6–2.0 μm in CS–1S^l^(1B). At 11 DPA, the average diameter of PB was 0.85 and 1.20 μm in CS and CS–1S^l^(1B), respectively. At 19 DPA, few PBs were detected in CS which was consistent with the fluorescence microscopy analysis result, whereas the diameter of PB was more than 2 μm in CS–1S^l^(1B) (Figure 7B). This result further verified that larger and more PBs in CS–1S^l^(1B) than that in CS.

SEM was used to analyze the starch changes in grain development in CS and CS–1S^l^(1B) (Figure S10). It is known that protein matrixes surround the starch granules in the mature wheat grains. In general, A-type starch granules were developed quickly from 5-15 DPA and B-type starch granules occured after 10 DPA in both lines. However the A-type starch granules in CS–1S^l^(1B) developed a little slower compared with those in CS in early stage, while neither A-type or B-type starch granules have obvious differences in the late stage.

## Discussion

### Molecular structures of 1S^l^-encoded HMW-GS and its relationships with gluten quality

It is well known that HMW glutenin subunits play an important role in determinating the elastic properties of glutenin complex [2]. In the current study, a pair of 1S^l^-encoded HMW-GS from *Aegilops longissima*, designated as 1S^l^×2.3*+1S^l^y16*, has been thoroughly characterized and studied (Figure 1 and Table 1). Molecular characterization showed that 1S^l^×2.3* had 941 amino acid (AA) residues with an extra 102 amino acid residue insertion in the central repetitive domain (603–704^th^), including 8 hexapeptides and 6 nonapeptides. The 1S^l^y16*, containing 749 amino acid residues, which appeared to be the largest y-type HMW subunit among the characterized y-type subunits so far.

A long repetitive domain is considered to have a positive influence on wheat flour quality [44]–[46] because it can form more β-turns structure conferring elasticity to the protein molecule [12], [19]. Insertion in the central repetitive domain could directly affect the functional properties [47], suggesting that the extra 102 amino acid residue insertion in the central repetitive domain of 1S^l^×2.3* subunit may play a positive role in dough visco-elastic properties. D'Ovidio and Anderson [48] suggested that y-type HMW-GS are among the main components responsible for differences in the technological characteristics of flour. It was deduced that the largest y-type subunit 1S^l^y16* could have positive effects on dough-making quality of the flour.

Theoretically, high glutamine content can stabilize the polymeric structure of glutenin through forming more hydrogen bonds [49] so that larger HMW-GS as well as LMW-GS rich in glutamines have a greater positive effect on dough strength than smaller subunits [50], [51]. It was found that the total glutamine content of 1S^l^×2.3*and 1S^l^y16* was similar with the subunit pair 1Dx5 + 1Dy10 that confers superior wheat flour processing qualities [4]. This may be another mechanism for the positive effects of 1S^l^×2.3* and 1S^l^y16*.

Another important factor to affect dough elasticity is the proportion of the consensus hexapeptides and nonapeptides in the repetitive domain. Masci *et al*. [46] reported that a rather large and regular repeated sequence domain is helpful in increasing the viscosity and elasticity of doughs through inter-molecular interactions. Higher proportion of repeats of the consensus type in 1Dy10 than that of 1Dy12, which may produce a more regular pattern of repetitive β-turns in the protein, contributed to better dough elasticity [20]. Compared to 1Dy10, 1S^l^y16*showed a similar repetitive domain, including 22 consensus hexapeptide (PGQGQQ) and 3 consensus (SGQGQQ) motifs. The 1S^l^×2.3*subunit had 23 consensus (PGQGQQ) and 8 consensus (SGQGQQ) hexapeptide motifs, as well as 24 tripeptide (consensus GQQ) motifs. Both subunits had no consensus nonapeptide motifs, suggesting that the consensus hexapeptides were more important than nonapeptides in determining the dough elasticity.

### HMW-GS expression and gluten quality

It has shown that the expression and accumulation patterns of storage proteins are associated with gluten quality properties [22], [52]. Higher accumulation of HMW-GS and LMW-GS at earlier grain developmental stage may contribute to the superior gluten quality of wheat [25]. The expression pattern of gliadin, LMW-GS and HMW-GS genes were similar and 10–18 DPA was the key dates of storage protein genes expression. The probable reason was that simultaneous storage protein can easily build up glutenin polymeric structure by the inter- or intra- molecular disulphide bonds [53]–[58]. Different storage proteins in the different cultivars may have different accumulation patterns, and different environmental conditions can also alter the accumulation rates, which consequently cause flour quality variations [56], [57], [59].

In the current study, more HMW-GS genes were transcribed in CS−1S^l^(1B), which takes shorter time to reach the maximum accumulation during grain development. This implies that good quality wheat may require more HMW-GS gene transcription at the mRNA level. Earlier synthesized HMW-GS mRNA may facilitate the accumulation of glutenin protein in advance, making an adequate time available to form the polymeric protein. In parallel of this, some enzymes (such as glutamine synthetase, PDI) at early development stages were highly active, which facilitate the folding of gluten proteins and formation of more regular glutenin polymers [25]. It was noteworthy that the HMW-GS genes were still transcribed in the late stage of grain development in CS–1S^l^(1B). This may result in more HMW-GS accumulation required for large glutenin polymer formations. The protein expression pattern was similar with the gene expression pattern at the mRNA level. More HMW-GSs and LMW-GSs were accumulated in CS-1S^l^(1B) than in CS according to the results of RP-HPLC (Figure 6). This suggested more glutenin transcription and expression in the good quality wheat than that of poor quality wheat.

### PDI affects bread wheat quality by forming disulfide bonds in glutenin polymeric proteins

The correct foldings of seed storage proteins were regulated by PDI proteins. It is logical to associate their expression with gluten quality conformation. In the current study, the overall expression pattern of *PDIL1-1* and *PDIL2-1* showed some similarities to HMW-GS genes and more *PDIL1-1* and *PDIL2-1* were expressed in CS–1S^l^(1B). They began to transcribe earlier than HMW-GS genes and their maximum expression point occurred earlier than that of *HMW-GS*. Earlier PDI gene transcription can facilitate the PDI protein expression to assist the HMW-GS accumulation and glutenin polymer formation during grain development. The expression of *PDIL1-1* and *PDIL2-1* was enhanced at the mid-stage of grain development, which enhanced the ability of inter-chain disulphide bonds formation at the later grain development. This phenomenon well explains the fact that a dramatic increase of large gluten polymers occurred and was stabilized by the formation and/or rearrangement of inter-chain disulphide bonds [60].

*PDIL4-1* is the only gene group that was encoded by group 1 chromosome, suggesting that some genes in this group were encoded by the introduced 1S^1^ chromosome. This makes it more important to study the expression difference of this gene group between CS and CS-1S^l^(1B). PDIL4-1 products contain two thioredoxin active domains that may be related to their role in the developing wheat endosperm. Transgentic *Arabidopsis* plants showed that PDIL4-1 involved in seed set and ovule development [37]. Higher expression of *PDIL4-1* was observed in CS–1S^l^(1B) than that in CS (Figure 5), which ensures efficient disulphide bond formation to synthesize glutenin polymeric protein. The four kinds of PDI and PDI like protein genes (*PDIL1-1*, *2-1*, *4-1*, *5-1*) expressed higher in the good quality CS-1S^l^(1B), indicating their functions related with the folding of seed storage proteins. These four types of PDIs are orthologous to four soybean PDI proteins which were proved to play a role in the folding of seed storage proteins [38].

Both PDIL3-1 and PDIL8-1 lacked the conserved glutamic acid proton acceptor in the N-terminal domains of and the cysteines in the active sites of the C-terminal [41]. The biological functions of *PDIL3-1* and *PDIL8-1* are still unknown. In this study, the expression pattern of these two gene groups was found to be similar in the early stage of grain development and did not show a significant expression difference between the two wheat lines. This may implies that these two genes are not involved in disulphide bond formation due to the lack of a few functional sites since the foreign chromosome in the substitution line did not have an impact on their expression behaviors. Although PDIL6-1 possessed both the -CXHC- active site and the conserved arginine residue, while its activity as a potential catalyst of oxidation reactions would be relatively inefficient because of the absence of the glutamic acid proton acceptor [61]. It was suggested that it may not involve storage protein folding and disulphide bond formation, because the expression of *PDIL6-1* was also similar in CS and CS–1S^l^(1B). The presence of a -CXHC- active site in combination with the three other determinants of enzymatic activity in PDIL7-1 and PDIL7-2 suggest that they would also be involved in disulphide bond formation at late-stage of grain development, especially they were all high expressed in the late stage of grain development of CS–1S^l^(1B).

### The development of PB in endosperm cells

The PBs developmental changes in different stage were in agreement with the previous studies [42], [43]. In our study, HMW-GS began to accumulate in protein bodies of micrometric size from 7 DPA. The fast accumulation of HMW-GS happened from 11 DPA to 19 DPA. In all the observed stages, PBs were larger in CS-1S^l^(1B) than that in CS. This suggests that HMW-GSs aggregates more in CS–1S^l^(1B) than that in CS, well consistent with the levels of HMW-GS gene transcription and translation. More HMW-glutenin accumulation leads to an increase amount of the macropolymer in gluten matrix and consequently leads to an increase of dough strength. An interesting phenomenon was found by SEM analysis: A-type starch granules developed rather slowly from 5 DPA to 15 DPA in CS-1S^l^(1B) when PBs grows very quickly. This suggested that the slow development A-type starch granules makes more space available for PBs enlargement and finally resulted in more HMW-GS accumulation in PBs present in the substitution line.

### Molecular mechanisms of superior breadmaking quality formation

**Figure 8:**
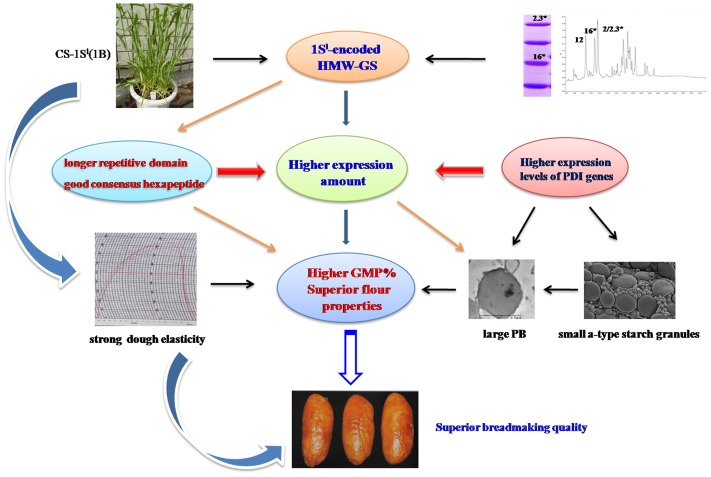
A putative molecular mechanism of HMW-GS from 1S^l^ genome positively affecting breadmaking quality.

Based on the above discussions, a hypothesis for deciphering the molecular mechanisms underlying the substitution line for superior bread-making quality can be proposed (Figure 8). Large HMW-GS genes with longer repetitive domain result in more α-helix and β-turns structure formation and these structures increase the dough strength. Higher properties of consensus repeats of hexapeptides in the repetitive domain may result in more regular pattern of repetitive β-turns in the protein, which contribute to the dough elasticity. In the substitution line, the y-type and x-type HMW-GS express in the similar level both at the mRNA and the protein levels (Figure 4). The y-type HMW-GS with more cysteines in N-terminal may help form more intermolecular disulfide bonds resulting in a larger glutenin polymer. Higher expression of *PDIL1-1*, *PDIL2-1*, *PDIL4-1*, *PDIL5-1* in early and mid-stage of grain development and *PDIL7-1* and *PDIL7-2* in late-stage of grain development play their roles to ensure correct glutenin polymer folding, while they also involve in starch accumulation and PB aggregation. Large PBs also mean more storage proteins and slow development of starch granules in the earlier grain development, which provides space requirement for PB enlargement.

Based on the above summary, the chromosome 1S^l^ from *Aegilops longissima* provides better dough strength than chromosome 1B. In the past, transgenic approach has been utilized to improve dough quality by transferring foreign glutenin genes into wheat [72]. However, our substitution line can be used to improve dough and baking qualities through non-transgenic approach, making the outcome be readily captured by consumers.

## Materials and Methods

### Plant materials

Chinese Spring (CS) and a CS substitution line viz. CS–1S^l^(1B) were used in this work. CS–1S^l^(1B) was developed in the Institute for Plant Breeding, Technische Universität München, Germany, in which the chromosome 1S^l^ from *Aegilops longissima* substituted 1B of CS [62]. The development procedures of CS–1S^l^(1B) were shown in Figure S1A. Briefly, CS was crossed with *Ae. longissima* and the F1 plants were generated from embryo rescue technique. CS-*Ae. longissima* amphiploid (42+14 = 56 chromosomes) was obtained after doubling the chromosome number by colchicine treatment. The amphiploid was backcrossed with CS for 6 times and an addition line (2n = 44, wheat + 1S^l^ chromosome pair) was obtained. The addition line was then crossed with CS monosomic line (CS mono 1B). The monosomic plants (2n = 41) were selected from progeny and the substitution line was developed after self pollination. The substitution line has similar agronomical characters with CS (Figure 1C, Table S1).

### Genome *in situ* hybridization

Genome *in situ* hybridization (GISH) was done to determine the chromosomes of CS-1S^l^(1B). The detailed process were referred to Han *et al*. [63] and mitotic chromosomes in CS-1S^l^(1B) were prepared from root tips using 45% acetic acid squash method. Total genomic DNA of *Ae. longssima* labeled with Dig-Nick translation mix according to the manufacturer's manual (Roche) was used as probe. Chromosomes were counterstained with 4′, 6-diamidino-2-6 phenylindole (DAPI) and observed using a fluorescent microscope (Olympus 7 BX61). Images were captured by a cooled CCD camera (CoolSnap fx, Photometrix).

### Gluten quality testing

Both CS-1S^l^(1B) and CS were planted in 2009 in the Beijing field trial station of the Chinese Academy of Agricultural Sciences. Three replicates of each line were included. Mean quality parameter value of the three replicates was taken. Gluten macropolymers (GMP) content was measured according to the protocol of Weegels *et al*. [17]. In short, grain sample (50 mg) was suspended in 1 ml of 1.5% SDS solution and centrifuged at 13,000 rpm for 30 min at 20°C. The nitrogen content of the sediment measured with biuret reagent was taken as the GMP content [64].

A 10-gram Mixograph (National Manufacturing) was used to evaluate the functional properties of CS-1S^l^(1B) dough based on the procedure outlined by Yan *et* *al*. [15]. The Mixograph assays and SDS-sedimentation value were carried out in three duplicates by following the 54–40A AACC method [65]. The extensogram method used was based on the ICC standard no. 114 [66]. Parameters of dough extensibility and dough maximum resistance (Rmax) were measured.

Bread baking experiment was also carried out to evaluate the breadmaking qualities of the two lines. The baking procedure used the standard rapid-mix-test with 1 kg flour at 14% moisture content. Each sample was mixed and baked in duplicate.

### Identification of seed proteins

#### Protein extraction

Based on solubility in a series of solvents, the albumins, globulins and gliadins were extracted from 15 mg CS and CS–1S^l^(1B) seeds by using 75 μl distilled water, 75 μl dilute salt solutions, and 120 μl 30% ethanol, respectively. The glutenins were extracted according to Yan *et al*. [31] by using commonly used glutenin extraction buffer (50% isopropanol, 80 mM Tris-HCl, pH 8.0) with 64.83 mM DTT and 1.4% 4-vinylpyridine (v/v).

#### SDS-PAGE

SDS-PAGE was performed with Bio-Rad PROTEAN II XL equipment based on a previously described method [31] with 12% gel and electrophoresed at 15 mA for 2 h.

#### RP-HPLC/UPLC

Reversed-phase high performance liquid chromatography (RP-HPLC) and reversed-phase ultra performance liquid chromatography (RP-UPLC) were used to separate HMW-GS and gliadins. The RP-HPLC system was Agilent 1100 combined with Zorbax 300SB-C18 columns [67]. Its solvents were composed of (A) water and (B) acetonitrile (ACN), both containing 0.06% (v/v) trifluoroacetic acid (TFA). The RP-UPLC (Waters Acquity UPLC^TM^, America) system was operated by strictly following the manufacturer's separation protocol (Waters instruction). Tunable ultraviolet (TUV) detector was used and 10 μl sample volume was injected for analyses.

#### SE-HPLC

Size exclusion-high performance liquid chromatography (SE-HPLC) was used to quantify the polymer glutenin contents in CS and CS–1S^l^(1B) according to the method of Rakszegia *et* *al*. [68]. A total of 10 mg flour was suspended in 1ml 0.5% (w/v) SDS in phosphate buffer (pH 6.9) and sonicated for 15 s. After centrifugation, the supernatant was filtered on a 0.45 μm PVDF filter. Analyses were performed on a Phenomenex BIOSEP-SEC 4000 column in an acetonitrile buffer of 0.05% (v/v) triflouroacetic acid and 0.05% (v/v) acetonitrile with a running time of 10 min (2 ml/min flow rate).

#### MALDI-TOF-MS and MALDI-TOF/TOF-MS

The procedure outlined by Gao *et al*. [67] was followed to conducted the MALDI-TOF-MS of HMW-GS. Sinapinic acid was used as matrix. The dried HMW-GS was dissolved in a mixture consisting of 0.05% TFA, 0.5% ACN, and H_2_O. The MALDI-TOF plate was loaded twice of 1 μl sample.

For MALDI-TOF/TOF-MS analysis, protein gels were visualized by a scanner with optics resolution setting at 300 dpi. Protein bands were excised manually and digested by trypsin. Tryptic peptides were analyzed with a MALDI-TOF mass spectrometer (SM, Shimadzu Biotech, Kyoto, Japan). All MS and MS/MS spectra were searched in the NCBI non-redundant green plant database MASCOT program using GPS Explorer™ software version 2.0 (Applied Biosystems).

### DNA extraction, PCR amplification and cloning

Genomic DNA was extracted from 50 mg leaves of CS–1S^l^(1B) seedlings 7 days after germination by using the cetytrimethylammonium bomide (CTAB) method as reported by Yan *et al*. [32]. For amplifying the complete open reading fragms (ORFs) of the HMW glutenin genes of CS–1S^l^(1B), two pairs of primers were designed by using Primer 5.0, including Sx1F/1R and Sy1F/1R that amplify the x-type and y-type HMW-GS genes, respectively (Table 5). The expected amplicons for both primer pairs include complete coding sequences plus partial upstream and downstream segments. The PCR amplification procedures were as following: an initial denaturation step at 94°C for 5 min followed by 34 cycles at 94°C for 45 s, 62°C for 80 s, 72°C for 150 s, and a final extension step at 72°C for 10 min. The PCR products were separated by 1% agrose gel and the amplified products of expected sizes were purified from the gel and cloned with pGEM-T plasmid vector (Promega). The recombined DNA clones were sequenced by TaKaRa Biotechnology (Dalian) CO., LTD, China. Each clone was sequenced three times for confirmation of clone sequences.

**Table 5:** List of primers for cloning and expressing the HMW-GS genes.

Primer name	Sequences of the primers
Sx1F	5′-CCTTCACTATCTCATCATCACCCAC-3′
Sx1R	5′-TAGGAGTCTGTTCGCATTCAGTGGC-3′
Sy1F:	5′-AATTTCATCATCACCCATAACAC CG-3′
Sy1R:	5′-ATTGGGTTTTACTCTAGTTACACG-3′
SxE1F	5′-AAACATATGACCGTCGCTGAAGGTGAGG-3′
SxE1R	5′-ACCGAATTCCTATCACTGGCTGGCCGAC-3′
SyE1F	5′-AAACATATGCTCAGCACCGCTGAAGGTGAGG-3′
SyE1R	5′-ACCGAATTCTCACTGGCTAGCCATCAATGCG-3′

### Sequence comparison, SNP/InDel identification and construction of phylogenetic tree

Software BioEdit 7.0 was used for sequence comparison and identification of single-nucleotide polymorphisms (SNPs) and insertions/deletions (InDels). And then Bioedit 7.0 was also used to perform the complete amino acid sequences multiple alignment of 2 cloned HMW-GS genes and 41 other genes from GenBank. Software MEGA4.1 [69] was used to construct phylogenetic trees. An evolutionary rate of 6.5×10^−9^ substitutions/site/year according to Allaby *et* *al*. [70] was used to estimate the divergent times among HMW-GS genes from different species.

### Heterologous expression and Western-blotting

The cloned HMW-GS genes were reamplified using expression PCR primers SxE1F/1R and SyE1F/1R for x- and y- type genes, resepectively (Table 5). Two restriction enzyme sites (NdeI and EcoRI) were added to the 5′ and 3′ end of the PCR product, respectively. The fragment was ligated into expression vector pET-28a (Novagen) and re-sequenced for confirmation of clone sequences, then transformed into an expression host *E. coli* strain BL21 (DE3) plysS. A polyclonal antibody specific for wheat HMW-glutenin subunits was produced by Beijing Genomic Institute (BGI) and the procedures of Western blotting were based on Yan *et al*. [15].

### mRNA extraction, cDNA synthesis and qRT-PCR

Developmental seeds from three spikes were combined together to extract total RNA by using Trizol extraction kit (Invitrogen) from endosperm of CS and CS-1S^l^(1B) at 5, 10, 15, 17, 19, 21, 23 and 25 days post anthesis (DPA) according to the manufacturer's instructions. Approximate 100 ng purified mRNA was used to synthesize cDNA with OligdT and random primer by using a superscript first-strand synthesis kit (Promega, Madison, WI, USA). The synthesized cDNA was used for quantitative real time-PCR (qRT-PCR). Two pairs of HMW-GS specific primers (specific for x-type and y-type HMW-GS genes) and 9 pairs of PDI and PDI-like genes specific primers were designed and used to measure the gene expression levels (Table S4). ADP-ribosylation factor (ADP-RF) was used as a control gene by using the primers ADPF and ADPR [71] (Table S4). All primer pairs were gene specific, through electrophoresis and unique melt peak verification. The efficiencies of all primer pairs ranging from 90% to 100% were determined by standard curves using a series of 10 DPA cDNA dilutions in CS. The qRT-PCR reactions were as following: an initial incubation of 94°C for 3 min, followed by 40 cycles of 94°C for 20 s, 58°C for 15 s, 72°C for 20 s. The gene expression data were processed and standardized according to CFX96 Real-Time system (Bio-Rad). Triplicate for each PCR reaction and at least three biological replicates were performed for each gene.

### Immunofluorescence microscopy and Electron micrographs

The developing grains in CS and CS–1S^l^(1B) were collected at 7, 11, 15, 19, 22 DPA, representing from relatively early to late stage. The seeds fixation, rinse, dehydration and thin sections preparation were based on Loussert *et* *al*. [43] with some modifications. HMW-GS Polyclonal antibody produced by BGI was used to conduct an immunofluorescence microscopy analysis. Endosperm sections fixed in the same conditions were used for both fluorescence microscopy and immunogold labeling transmission electron microscope (TEM). For TEM analysis, the procedures were according to Loussert *et* *al*. [43] with the second antibody being 15 nm gold conjugated goat anti-rabbit IgG.

Grain endosperms at different developmental stages (5, 10, 15, 20, 25 DPA) were examined by scanning electron microscopy (SEM). They were immediately fixed in a solution containing 44.5% ethanol, 1.85% methanal and 6% glacial acetic acid for 60 min followed by an overnight treatment at 4°C, then transferred into 70% ethanol and stored at 4°C prior to analysis. Samples were dehydrated sequentially through an ethanol concentration series (50%, 70%, 85%, 95% and 100%v/v, with 1 h incubation in each solution) and thoroughly dried before examination. Finally they were observed with a scanning electronic microscope (SEM) S-4800 FESEM (Hitachi, Japan).

## Supporting Information

Figure S1 **The procedure of producing the CS-1S^l^(1B) (A), cytological characterization of the CS-1S^l^(1B) by GISH (B) and the morphological characterizations of plants, spikes and seeds of CS and the substitution line (C).** GISH was performed using genomic DNA of *Ae*. *longissima*, indicating presence of 1S^l^ chromosomes of *Ae*. *longissima* (shown in red colour).
(TIF)

Figure S2 **The compositions of glutenins and gliadins in CS and CS-1S^l^(1B) identified by RP-HPLC and RP-UPLC.** A and B: Glutenin compositions in CS and the substitution line by RP-HPLC and RP-UPLC, respectively. C: Gliadin compositions identified by RP-HPLC. Different HMW-GS, LMW-GS and gliadins from CS and CS-1S^l^(1B) were indicated.
(TIF)

Figure S3 **The compositions of albumins and globulins (Agl) in CS and CS-1S^l^(1B) identified by SDS-PAGE.**
(TIF)

Figure S4 **Mutliple sequence alignment of the derived amino acid sequences of 1Bx-type HMW-GS genes.** The alignments were assembled by eye to demonstrate the repeat structure of the central domain. Hexapeptide repeat motifs are boxed in green color and nonapeptide repeat motifs are boxed in red color. N-terminal, Repetitive domain and C-terminal of HMW-GS were marked, respectively.
(TIF)

Figure S5 **Mutliple sequence alignment of the derived amino acid sequences of 1By-type HMW-GS genes.** The alignments were assembled by eye to demonstrate the repeat structure of the central domain. Hexapeptide repeat motifs are boxed in green color and nonapeptide repeat motifs are boxed in red color. N-terminal, Repetitive domain and C-terminal of HMW-GS were marked, respectively.
(TIF)

Figure S6 **Identification of the expressed HWM-GS (with deleted the signal domain) in** ***E*** **.** ***coli*** **by SDS-PAGE and Western blotting.** a, b, pET-28a represent expression protein of pET-2.3*, pET-16* and pET-28a plasmid clone, respectively. CS-1S^l^(1B) represents the glutenins in CS-1S^l^(1B). The expressed proteins were indicated by red arrow.
(TIF)

Figure S7 **Phylogenetic tree constructed based on the complete amino acid sequences of 41 HMW-GSs by MEGA4.1.**
(TIF)

Figure S8 **The expression profile of four groups of PDI and PDI like genes (** ***PDIL3-1*** **,** ***6-1*** **,** ***7-1*** **,** ***7–2*** **and** ***8–1*** **) in CS and CS-1S^l^(1B) by qRT-PCR.**
(TIF)

Figure S9 **Quantification of glutenin content by SE-HPLC in CS and CS-1S^l^(1B).**
(TIF)

Figure S10 **SEM profiles of grains at five developmental stages (5, 10, 15, 20 and 25 DPA) in CS and CS-1S^l^(1B).** A and B starch granules are indicated by arrows.
(TIF)

Table S1 **Comparsion of agronomical characters between CS and CS-1S^l^(1B).**
(DOCX)

Table S2 Identification of two HMW glutenin subunits (1S^l^x 2.3* and 1S^l^y16*) in CS-1S^l^(1B) by MALDI-TOF/TOF-MS.
(DOCX)

Table S3 **The average area of protein bodies labeling with anti-HMW-GS in 10^4^μm^2^ of CS and CS-1S^l^(1B) from 7-22 DPA of grain development.**
(DOCX)

Table S4 **List of primers used for qRT-PCR of HMW-GS, PDI and PDI-like genes.**
(DOCX)
