# Correction: Molecular Mechanisms of HMW Glutenin Subunits from 1S^l^ Genome of *Aegilops longissima* Positively Affecting Wheat Breadmaking Quality

**DOI:** 10.1371/annotation/d86a2c61-0953-4555-95bc-1ae9b8e3452c

**Published:** 2013-06-27

**Authors:** Shunli Wang, Zitong Yu, Min Cao, Xixi Shen, Ning Li, Xiaohui Li, Wujun Ma, H. Weißgerber, Friedrich Zeller, Sai Hsam, Yueming Yan

The number of one of the grant from the first funding body, National Natural Science Foundation of China, is incorrect. The correct grant numbers from this funding body are: 30830072, 31271703. 

