# Supplementary figures and images for: Molecular Mechanisms of HMW Glutenin Subunits from 1Sl Genome of Aegilops longissima Positively Affecting Wheat Breadmaking Quality
Source: PLoS One. 2013 Apr 4;8(4):e58947. doi: 10.1371/journal.pone.0058947 (PMC3617193; doi:10.1371/journal.pone.0058947)

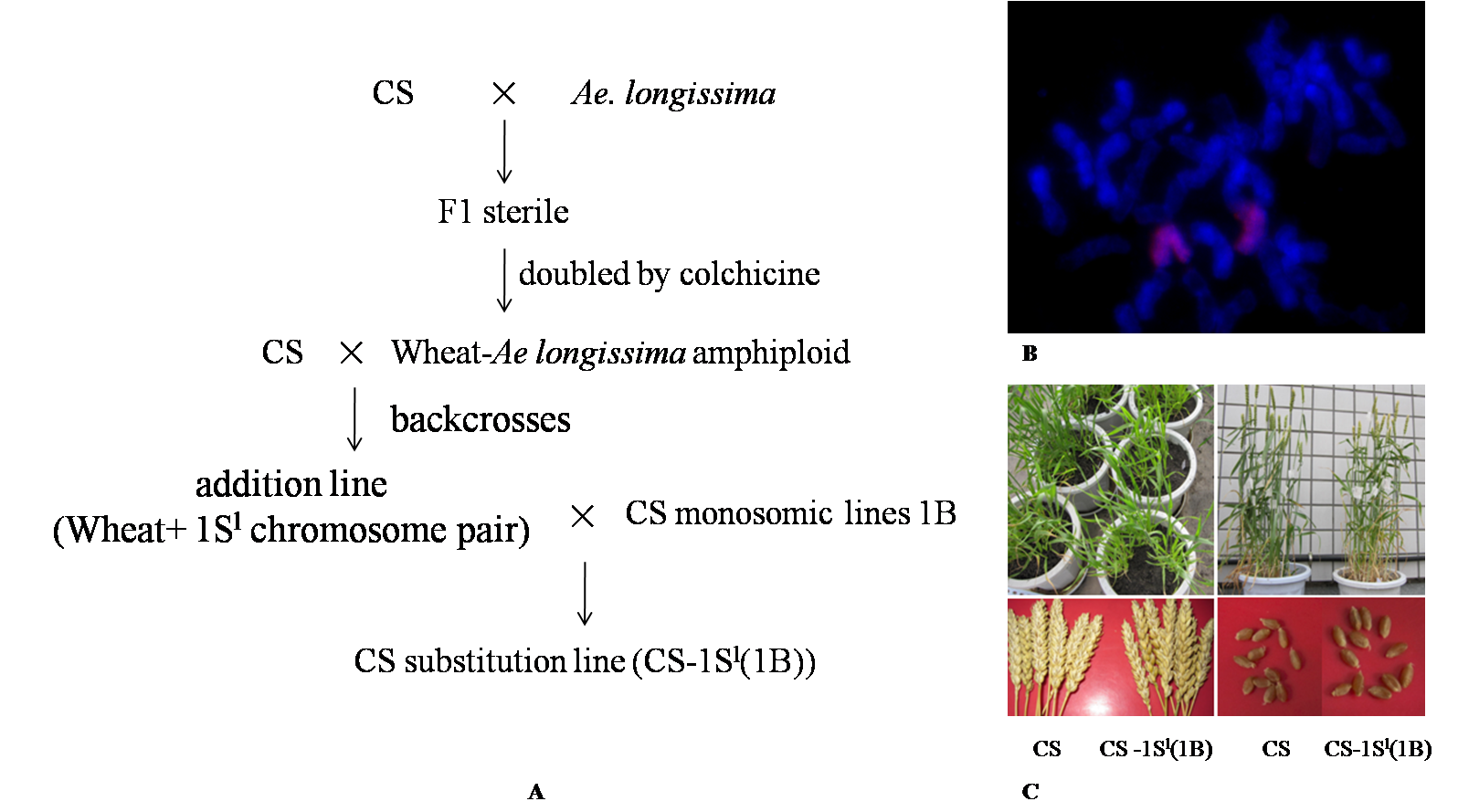

Supplement: Figure S1 — The procedure of producing the CS-1Sl(1B) (A), cytological characterization of the CS-1Sl(1B) by GISH (B) and the morphological characterizations of plants, spikes and seeds of CS and the substitution line (C). GISH was performed using genomic DNA of Ae. longissima, indicating presence of 1Sl chromosomes of Ae. longissima (shown in red colour). (TIF) [file pone.0058947.s001.tif]

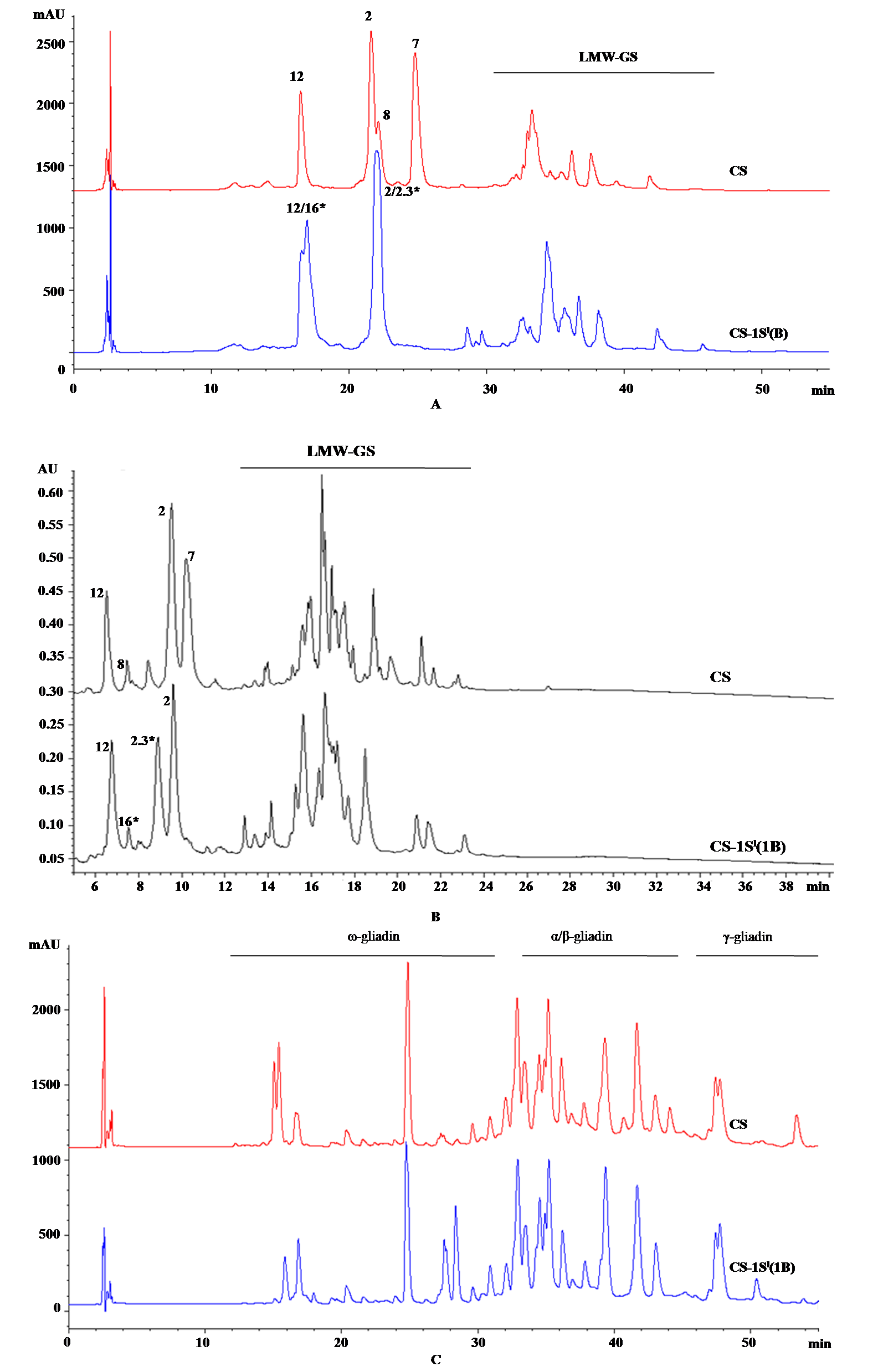

Supplement: Figure S2 — The compositions of glutenins and gliadins in CS and CS-1Sl(1B) identified by RP-HPLC and RP-UPLC. A and B: Glutenin compositions in CS and the substitution line by RP-HPLC and RP-UPLC, respectively. C: Gliadin compositions identified by RP-HPLC. Different HMW-GS, LMW-GS and gliadins from CS and CS-1Sl(1B) were indicated. (TIF) [file pone.0058947.s002.tif]

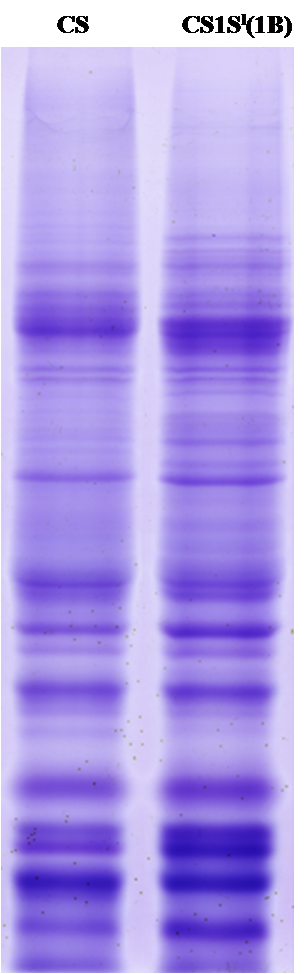

Supplement: Figure S3 — The compositions of albumins and globulins (Agl) in CS and CS-1Sl(1B) identified by SDS-PAGE. (TIF) [file pone.0058947.s003.tif]

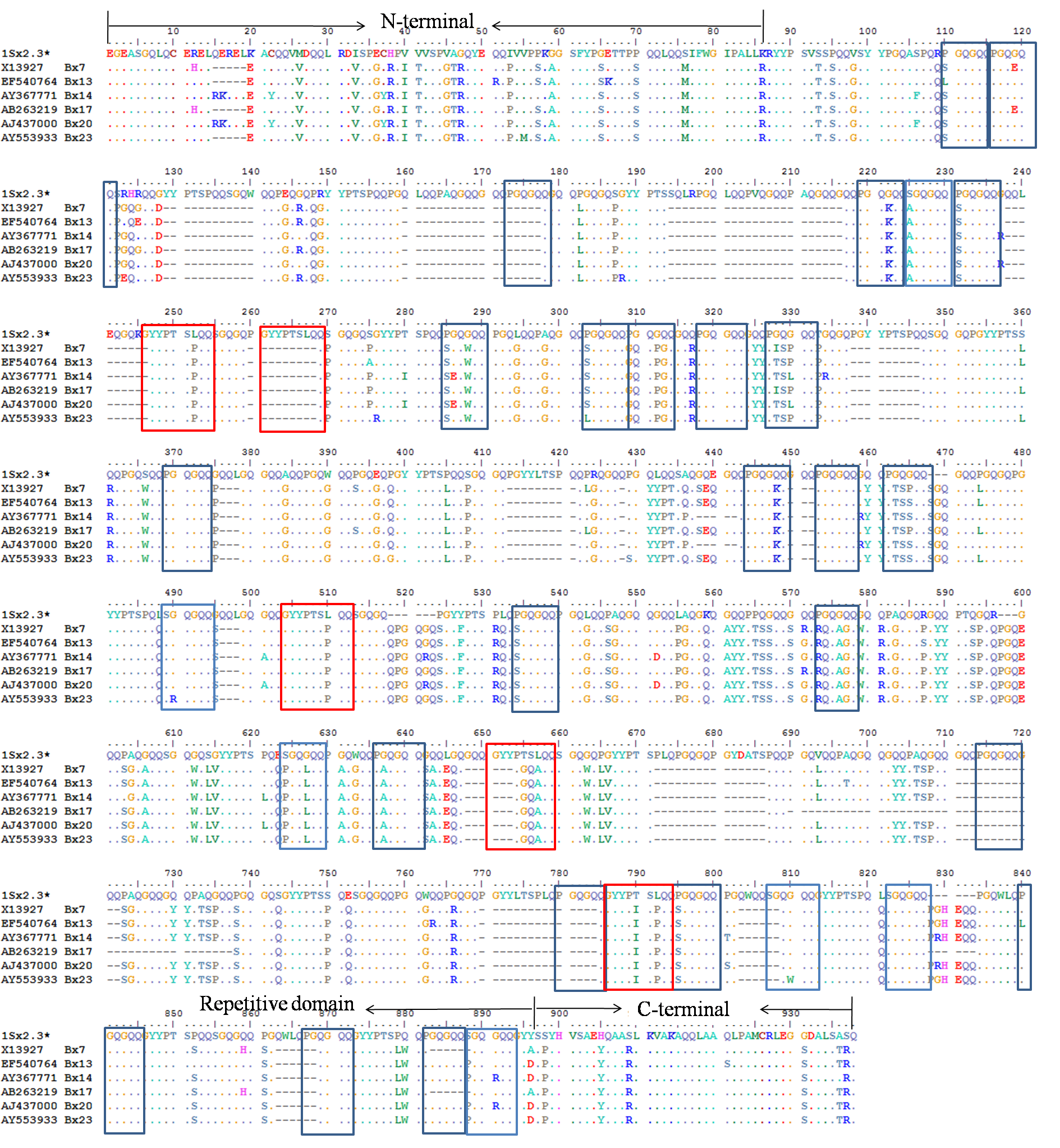

Supplement: Figure S4 — Mutliple sequence alignment of the derived amino acid sequences of 1Bx-type HMW-GS genes. The alignments were assembled by eye to demonstrate the repeat structure of the central domain. Hexapeptide repeat motifs are boxed in green color and nonapeptide repeat motifs are boxed in red color. N-terminal, Repetitive domain and C-terminal of HMW-GS were marked, respectively. (TIF) [file pone.0058947.s004.tif]

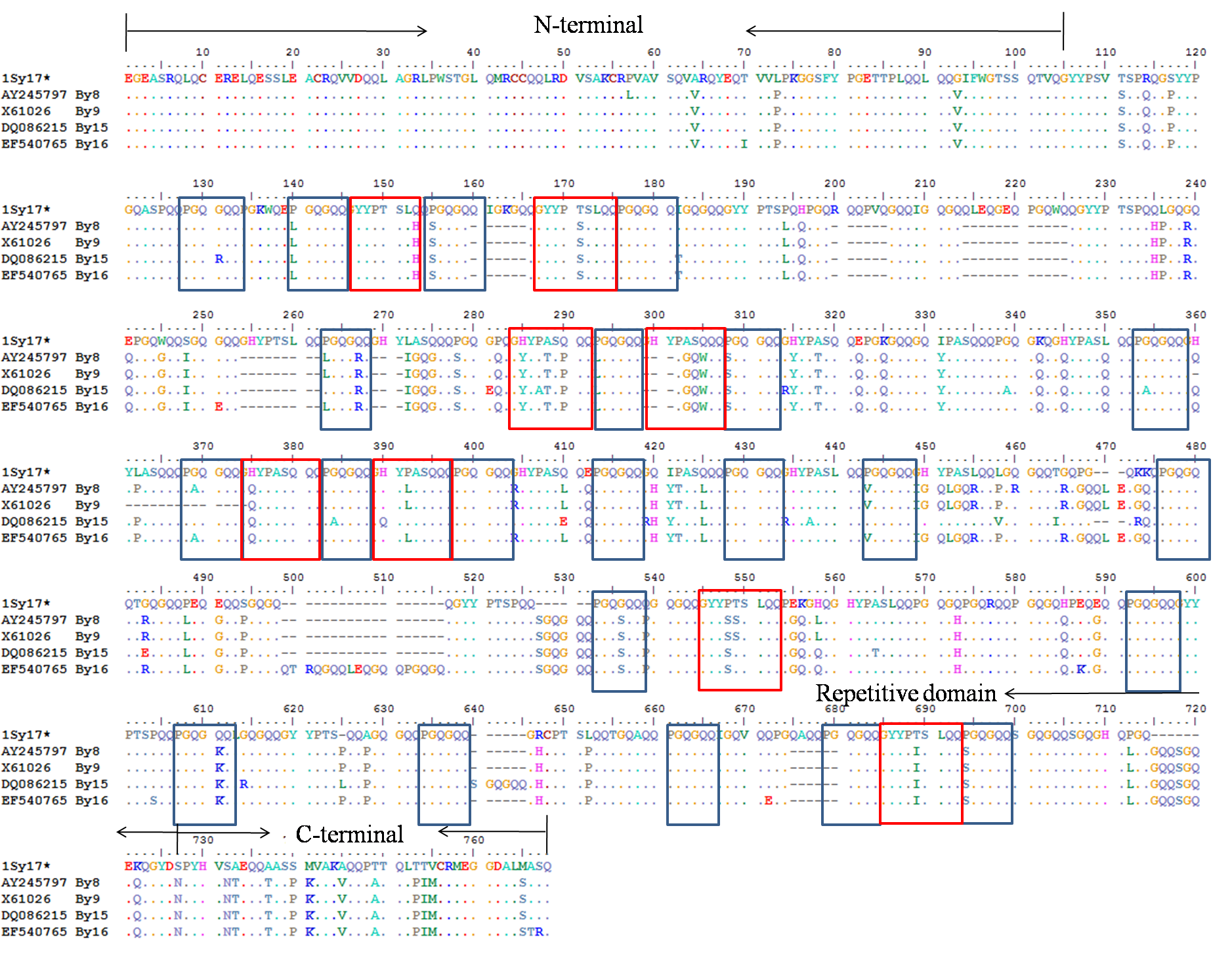

Supplement: Figure S5 — Mutliple sequence alignment of the derived amino acid sequences of 1By-type HMW-GS genes. The alignments were assembled by eye to demonstrate the repeat structure of the central domain. Hexapeptide repeat motifs are boxed in green color and nonapeptide repeat motifs are boxed in red color. N-terminal, Repetitive domain and C-terminal of HMW-GS were marked, respectively. (TIF) [file pone.0058947.s005.tif]

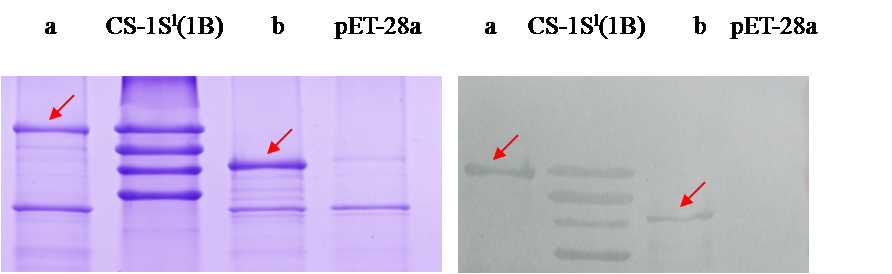

Supplement: Figure S6 — Identification of the expressed HWM-GS (with deleted the signal domain) in E . coli by SDS-PAGE and Western blotting. a, b, pET-28a represent expression protein of pET-2.3*, pET-16* and pET-28a plasmid clone, respectively. CS-1Sl(1B) represents the glutenins in CS-1Sl(1B). The expressed proteins were indicated by red arrow. (TIF) [file pone.0058947.s006.tif]

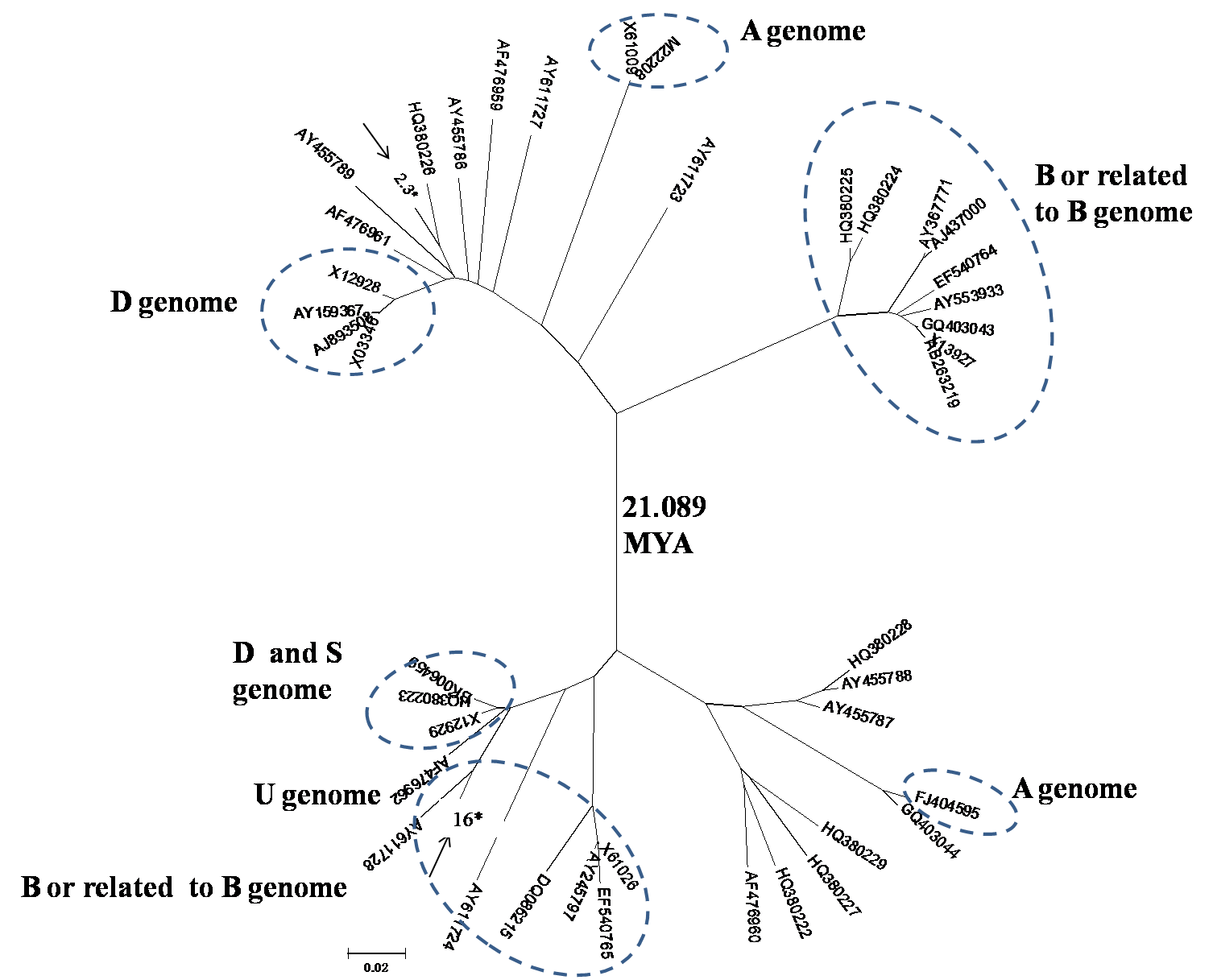

Supplement: Figure S7 — Phylogenetic tree constructed based on the complete amino acid sequences of 41 HMW-GSs by MEGA4.1. (TIF) [file pone.0058947.s007.tif]

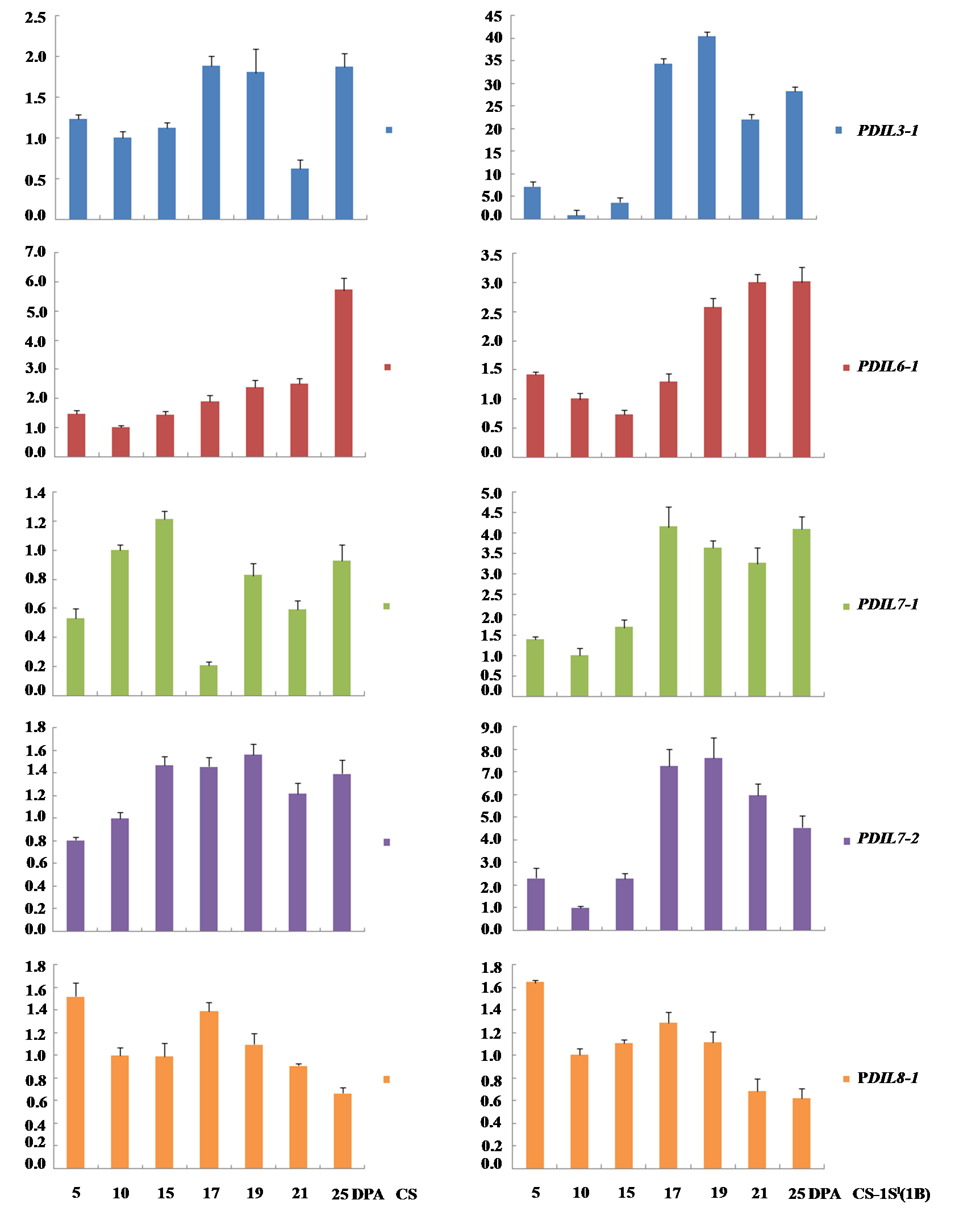

Supplement: Figure S8 — The expression profile of four groups of PDI and PDI like genes ( PDIL3-1 , 6-1 , 7-1 , 7–2 and 8–1 ) in CS and CS-1Sl(1B) by qRT-PCR. (TIF) [file pone.0058947.s008.tif]

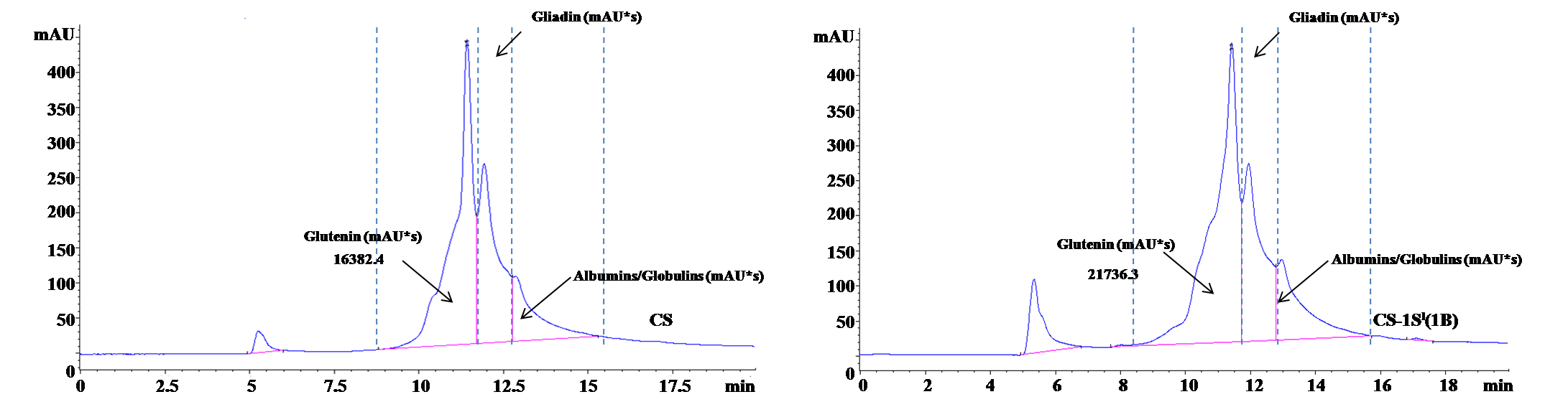

Supplement: Figure S9 — Quantification of glutenin content by SE-HPLC in CS and CS-1Sl(1B). (TIF) [file pone.0058947.s009.tif]

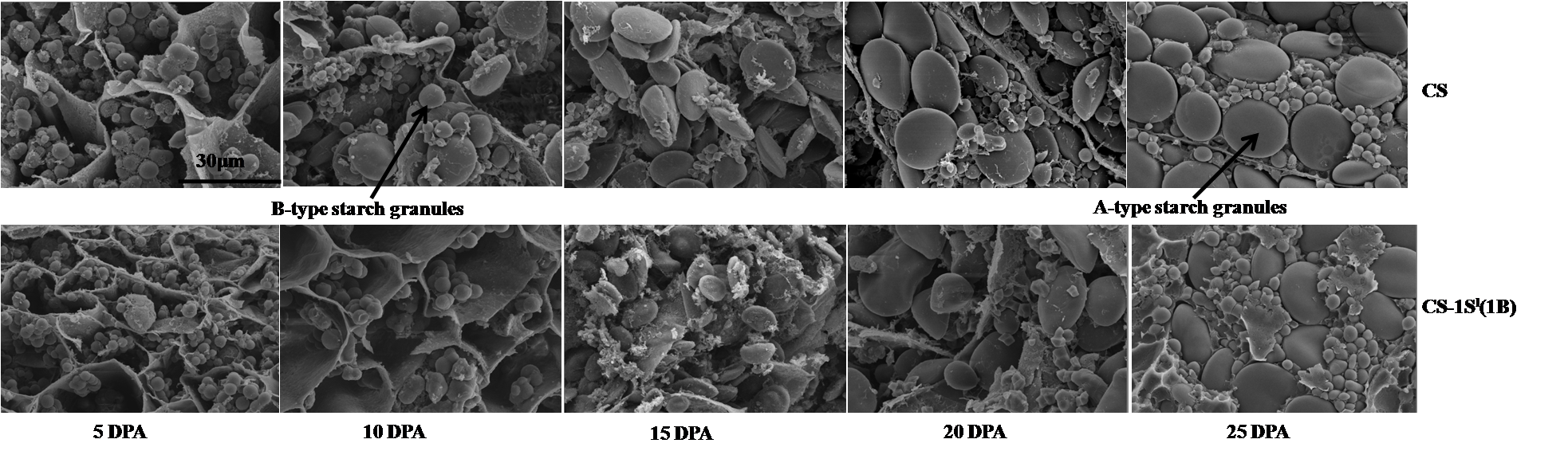

Supplement: Figure S10 — SEM profiles of grains at five developmental stages (5, 10, 15, 20 and 25 DPA) in CS and CS-1Sl(1B). A and B starch granules are indicated by arrows. (TIF) [file pone.0058947.s010.tif]
