# Supplementary material for: Molecular Mechanisms of HMW Glutenin Subunits from 1Sl Genome of Aegilops longissima Positively Affecting Wheat Breadmaking Quality
Source: PLoS One. 2013 Apr 4;8(4):e58947. doi: 10.1371/journal.pone.0058947 (PMC3617193; doi:10.1371/journal.pone.0058947)
Supplement: Table S1 — Comparsion of agronomical characters between CS and CS-1Sl(1B). (DOCX) [file pone.0058947.s011.docx]

Supporting Tables:

**Table S1** Comparsion of agronomical characters between CS and CS-1S^l^(1B)

| Lines | Height (cm) | Length of spike | Grain number  per spike | Thousand grain  weight (g) |
| --- | --- | --- | --- | --- |
| CS | 70.76±3.94 | 6.40±1.22 | 54.2±3.19 | 21.83±0.07 |
| CS1S^l^ (1B) | 69.85±2.18 | 6.55±0.69 | 54.5±2.91 | 25.97±1.44 |
