# Supplementary material for: Molecular Mechanisms of HMW Glutenin Subunits from 1Sl Genome of Aegilops longissima Positively Affecting Wheat Breadmaking Quality
Source: PLoS One. 2013 Apr 4;8(4):e58947. doi: 10.1371/journal.pone.0058947 (PMC3617193; doi:10.1371/journal.pone.0058947)
Supplement: Table S2 — Identification of two HMW glutenin subunits (1Slx 2.3* and 1Sly16*) in CS-1Sl(1B) by MALDI-TOF/TOF-MS. (DOCX) [file pone.0058947.s012.docx]

**Table S2** Identification of two HMW glutenin subunits (1S^l^x 2.3* and 1S^l^y16*) in CS-1S^l^(1B) by MALDI-TOF/TOF-MS

| HMW-GS | Protein identified | Species | Accession no. | Tryptic Fragments identified by MS data | position | Matched pep.^a^ | Protein Score | Protein Score C. I. % | Total Ion | Total Ion C.I.% |
| --- | --- | --- | --- | --- | --- | --- | --- | --- | --- | --- |
| 1S^l^x2.3* | x- type HMW-GS | *Aegilops bicornis* | gi\|47834185 |  |  | 3 | 159 | 100 | 155 | 100 |
|  |  |  |  | ACQQVMDQQLR | 42-52 |  |  |  |  |  |
|  |  |  |  | AQQLAAQLPAMCR | 965-997 |  |  |  |  |  |
|  |  |  |  | QQQPEGQYGYPQTPSRSQQPGQWQ | 141-164 |  |  |  |  |  |
| 1S^l^y16* | y- type HMW-GS | *T. aestivum* | gi\|24474920 |  |  | 6 | 104 | 100 | 63 | 99.96 |
|  |  |  |  | CCQQLR | 65-70 |  |  |  |  |  |
|  |  |  |  | QVVDQQLAGR | 45-54 |  |  |  |  |  |
|  |  |  |  | LPWSTGLQMR | 55-64 |  |  |  |  |  |
|  |  |  |  | QYEQTVVPPK | 87-96 |  |  |  |  |  |
|  |  |  |  | GKQGYYPTSLQQ | 185-196 |  |  |  |  |  |
|  |  |  |  | QGGQSQYPYGPKGQASPQQPGQ | 136-157 |  |  |  |  |  |

a. Multiple matches to peptides with the same primary sequence count.
