# Supplementary material for: Molecular Mechanisms of HMW Glutenin Subunits from 1Sl Genome of Aegilops longissima Positively Affecting Wheat Breadmaking Quality
Source: PLoS One. 2013 Apr 4;8(4):e58947. doi: 10.1371/journal.pone.0058947 (PMC3617193; doi:10.1371/journal.pone.0058947)
Supplement: Table S3 — The average area of protein bodies labeling with anti-HMW-GS in 104μm2 of CS and CS-1Sl(1B) from 7-22 DPA of grain development. (DOCX) [file pone.0058947.s013.docx]

**Table S3** The average area of protein bodies labeling with anti-HMW-GS in 10^4^μm^2^ of CS and CS-1S^l^(1B) from 7-22 DPA of grain development.

| CS | PBs | 7 | 11 | 15 | 19 | 22 DPA |
| --- | --- | --- | --- | --- | --- | --- |
|  | PB <1μm | 18.73±0.90 | 43.63±5.05 | 10.20±1.11 | 10.67±2.38 | 2.64±0.64 |
|  | 1μm<PB<5μm | 6.19±0.95 | 9.58±1.90 | 2.86±0.39 | 0.76±0.04 | 0.33±0.05 |
|  | PB>5μm | 1.17±0.23 | 0.47±0.55 | 0.89±1.07 | 0±0 | 0.33±0.37 |
|  | Total | 26.09 | 53.68 | 13.95 | 11.43 | 3.30 |
| CS-1S^l^(1B) | PB <1 μm | 8.54±0.16 | 31.964±0.22 | 26.924±0.92 | 35.714±1.82 | 6.70±0.98 |
|  | 1μm<PB<5μm | 3.14±0.08 | 7.03±0.38 | 5.00±0.32 | 10.54±0.76 | 1.47±0.12 |
|  | PB>5μm | 1.00±0.05 | 1.46±0.21 | 2.31±0.23 | 2.86±0.28 | 0.42±0.08 |
|  | Total | 12.68 | 40.45 | 34.23 | 49.11 | 8.59 |
