# Supplementary material for: Molecular Mechanisms of HMW Glutenin Subunits from 1Sl Genome of Aegilops longissima Positively Affecting Wheat Breadmaking Quality
Source: PLoS One. 2013 Apr 4;8(4):e58947. doi: 10.1371/journal.pone.0058947 (PMC3617193; doi:10.1371/journal.pone.0058947)
Supplement: Table S4 — List of primers used for qRT-PCR of HMW-GS, PDI and PDI-like genes. (DOCX) [file pone.0058947.s014.docx]

**Table S4** List of primers used for qRT-PCR of HMW-GS and PDI and PDI-like genes

| Genes name | Forward | Reverse |
| --- | --- | --- |
| *HMW-X* | 5’-ATGTTAGCGCGGAGCACCAG-3’ | 5’-CTATCACTGGCTGGCCGACA-3’ |
| *HMW-Y* | 5’-CAAGGCTACGACAGCCCATAC-3’ | 5’-CACCCTCCATCCGACACACT-3’ |
| *PDIL1-1* | 5’-GGCTGGCCAAATTGTTGCT-3’ | 5’-CGGACTTCCTGAATGGTGTCA-3’ |
| *PDIL2-1* | 5’-TCCCCACGATACTCTTCTATCCA-3’ | 5’-CGGTCCCCCTCGAAAGTTAT-3’ |
| *PDIL3-1* | 5’-CATTTGCTCTTGGCTTATTC-3’ | 5’-CTCTGGTTCGCTTTTCAC-3’ |
| *PDIL4-1* | 5’-GTGGTCCTGCTGTCAAGCAT-3’ | TCAGAGCCCTTCTGCAATATC-3’ |
| *PDIL5-1* | 5’-GAAATCACCGAGTTTGTGAAGGA-3’ | 5’-TCCCATGGTTCTGACTCTACGA-3’ |
| *PDIL6-1* | 5’-ACCGCAATCAACTTTTCC-3’ | 5’-TTCTGCGTCCCTTGGTG-3’ |
| *PDIL7-1* | 5’-ATGCGAACCGTGATTTGG-3’ | 5’-ATTGGGATGCTTGGTCGC-3’ |
| *PDIL7-2* | 5’-ATTCCAGCATTGGTTTCGC-3’ | 5’-GAAGCAGGGATTGCCGTAT-3’ |
| *PDIL8-1* | 5’-TGTGCTATTATTGGTGGAGTTTTCA-3’ | 5’-AGCGTGTTGTGCAAGATGGA-3’ |
| *ADP-RF* | 5’- GCTCTCCAACAACATTGCCAAC -3’ | 5’- GCTTCTGCCTGTCACATACGC -3’ |
